# Disturbance of cytoskeleton induced by ligustilide promotes hepatic stellate cell senescence and ameliorates liver fibrosis

**DOI:** 10.7150/thno.108869

**Published:** 2025-07-24

**Authors:** Jiaorong Qu, Jianan Li, Le Wang, Yufei Li, Yinqiang Zhang, Jingtao Li, Zixuan Huo, Junsong Han, Runping Liu, Guifang Fan, Yinhao Zhang, Xiaoyong Xue, Xiaojiaoyang Li

**Affiliations:** 1 School of Life Sciences, Beijing University of Chinese Medicine, 11 Bei San Huan Dong Lu, Beijing, 100029, China.; 2 School of Chinese Materia Medica, Beijing University of Chinese Medicine, 11 Bei San Huan Dong Lu, Beijing, 100029, China.; 3 Xiyuan Hospital, China Academy of Chinese Medical Sciences, Beijing, 100091, China.; 4 Departments of Infectious Disease, The Affiliated Hospital of Shanxi University of Chinese Medicine, Xian, 712000, China.; 5 Dongzhimen Hospital, Beijing University of Chinese Medicine, 11 Bei San Huan Dong Lu, Beijing, 100029, China.

**Keywords:** ligustilide, cytoskeleton, YAP, cGAS-STING, senescence

## Abstract

**Background and aims:** Inducing the senescence of activated hepatic stellate cells (HSCs) has emerged as a promising therapeutic strategy for liver fibrosis, with potential connections to the Yes-associated protein (YAP)-controlled cGAS-STING pathway. However, the regulatory role of cytoskeletal dynamics on HSC senescence and its potential as a target for natural products have remained poorly understood.

**Methods:** We employed preclinical *in vivo* and *in vitro* transcriptome analyses, experimental systems, Tmem173^-/-^ mice and liver-specific STING knockdown mice to demonstrate the anti-fibrotic effects and mechanism of ligustilide (LIG).

**Results:** LIG selectively bound to monomeric globular actin (G-actin), thereby preventing its polymerization into polymeric filamentous actin (F-actin), which disturbed its interaction with intermediate filament component lamin A/C and initially destroyed the nuclear membrane. Moreover, the disruption of nuclear membrane caused YAP leakage from nuclear, which in turn suppressed lamin A/C and created a deleterious feedback loop that exacerbated nuclear membrane destabilization. Consequently, nuclear double stranded DNA (dsDNA) leakage caused by the above damage cascade ultimately triggered the activation of the cGAS-STING signaling pathway, promoting senescence-associated secretory phenotypes (SASPs) release and inducing HSC senescence. Moreover, the induction of HSC senescence and anti-fibrotic effects of LIG were completely abrogated in both whole-body STING knockout and liver-specific STING knockdown mice.

**Conclusions:** By interacting with G-actin, LIG disrupted the cytoskeleton to compromise nuclear integrity with the involvement of YAP and further stimulated the cGAS-STING pathway, leading to the release of SASPs and HSC senescence, which ultimately mitigated liver fibrosis.

## Introduction

Liver fibrosis is characterized by the excessive deposition of extracellular matrix (ECM), which progressively restricts normal liver regeneration and contributes to a persistent fibrotic microenvironment. During liver fibrogenesis, quiescent hepatic stellate cells (HSCs) transition to an activated phenotype, where they produce ECM proteins, which are considered the core driving factor of the disease 1. Hence, either eliminating activated HSCs or reversing their activation state represents a promising strategy for treating liver fibrosis. Although research on antifibrotic drugs has focused predominantly on inhibiting the proliferation of activated HSCs and inducing their apoptosis, the outcomes of current treatments remain unsatisfactory, necessitating alternative strategies that target HSCs 2. Cellular senescence is a biological process in which cells permanently stop dividing and enter a state of growth arrest. Senescence is widely accepted to be associated with the inhibition of the cell cycle-related proteins and release of secretion factors associated with the senescence-associated secretory phenotypes (SASP) that facilitate the recognition and clearance of aged HSCs from the liver 3. Recent studies have demonstrated that senescent HSCs lose their activated phenotype and profibrotic function 4, suggesting that inducing HSC senescence is another highly promising emerging strategy for targeting and eliminating activated HSCs.

The mechanisms underlying HSC senescence have been extensively characterized in recent studies 5, 6. The accumulation of cytoplasmic DNA, including cytoplasmic chromatin fragments, mitochondrial DNA and cytosolic DNA, in HSCs activates the cyclic-GMP-AMP synthase (cGAS)-stimulator of interferon gene (STING) pathway and promotes the production and release of secretory factors associated with the SASP, thereby promoting HSC senescence 7. Furthermore, Yes-associated protein (YAP) has been identified as a critical factor determining whether HSCs remain activated or enter a state of senescence 4. While YAP has been shown to inhibit cGAS-STING-mediated senescence in stromal cells 8, its role in regulating HSC senescence remains unexplored. The YAP and cGAS-STING pathway have emerged as targets for HSC senescence, however, these downstream effectors are ultimately controlled by undefined upstream modulators. As reported, ECM remodeling and cell cytoskeleton have been recently demonstrated to be closely related to viscoelastic multiscale mechanical indexes, which can be used to assess liver fibrosis and treatment outcomes 9, 10. Recently, we reported that the remodeling of F-actin directly modulates disease processes 11. During fibrosis, a stiffened ECM activates microfilament rearrangement and actomyosin contractility, disrupting mechanotransduction pathways and organelle homeostasis, which includes increased mitochondrial ROS production, the translocation of transcriptional factors, and loss of endoplasmic reticulum (ER) calcium homeostasis, Golgi-related secretory pathways and perturbed multivesicular endosomes, which aggravated liver fibrosis 12. Notably, maintaining the homeostatic equilibrium of F-actin is crucial for preventing cellular senescence. In contrast, disruption of microfilament rearrangement can result in several detrimental consequences, such as mitochondrial dysfunction, collapse of the nuclear cytoplasmic bridge and nuclear envelope damage, which drives cellular senescence 13, 14. Additionally, the microfilament governs the nucleocytoplasmic shuttling of YAP through polymerization and depolymerization 15. Interestingly, our previous work revealed that the remodeling of microfilaments in activated HSCs is dynamic and plays a significant role in guiding the cell fate of HSCs, thereby greatly contributing to the process of liver fibrosis 16. These findings suggest that microfilaments could be a powerful upstream regulators of HSC senescence. However, the mechanisms underlying the alterations in the microfilaments associated with HSC senescence are largely unknown.

Natural products have attracted significant attention as potential therapeutics for liver fibrosis 17. Studying the development of effective antifibrotic drugs is of paramount clinical importance for both the prevention and treatment of fibrosis. Ligustilide (LIG), a phthalide compound present in plants such as *Chuanxiong Rhizoma* (Chuanxiong) and *Angelicae Sinensis Radix* (Danggui), has emerged as a novel compound with the potential to modulate cellular senescence 18, demonstrating its promising effects on age-associated neurological disorders 19, 20. Moreover, LIG was recently demonstrated to markedly ameliorate alcoholic liver steatosis and hepatocellular carcinoma, underscoring its potential hepatoprotective properties 21.

## Methods

### Reagents

LIG was purchased from MedChemExpress (HY-N0401, Shanghai, China). Ursodeoxycholic acid (UDCA) was purchased from Aladdin (U110695, Shanghai, China). Jasplakinolide (JAS) was purchased from Santa Cruz Biotechnology (SC-202191A, Shanghai, China). H-151 was purchased from Shanghai yuanye Bio-Technology Co.,Ltd (S89040, Shanghai, China). Transforming growth factor (TGF)-β was purchased from Novoprotein (CA59, Nanjing, China). anti-DNA Antibody (CBL186, Millipore, Bedford, MA, USA).

### Animal experiments

All animal studies along with procedures were approved by the Institutional Animal Care and Use Committee of Beijing University of Chinese Medicine. C57BL/6J mice (8 weeks old, 24-25 g, male) were purchased from SIBEIFU Biotechnology Co, Ltd. in Beijing, China. Normally, mice were bred in a room at 25 ℃ with a cycle of 12 h light/12 h dark and fed with an unrestricted chow diet and water during the whole research. As UDCA is the first-line treatment for primary biliary cholangitis (PBC) 22, we applied UDCA as the positive control drug. After feeding for 1 week, mice were randomly divided into sham, bile duct ligation (BDL), BDL + 5 mg/kg LIG, BDL + 10 mg/kg LIG, BDL + 20 mg/kg LIG and BDL + 40 mg/kg UDCA, n = 6. Firstly, mice were pre-administered with various doses of LIG or UDCA (i.p.) for 2 days prior to BDL. After surgery, animals entered a 2-day postoperative recovery phase without drug intervention. Therapeutic administration (i.p.) with escalating LIG doses or UDCA was then initiated and maintained for 4 additional days. All animals in the groups were sacrificed 7 days after BDL surgery, and their sera and liver tissues were collected.

To detect the efficacy of LIG for normal mice, mice were randomly divided into 4 groups: (1) control; (2) 5 mg/kg LIG; (3) 10 mg/kg LIG; (4) 20 mg/kg LIG. The dosage and timing of the medication are the same as that in the above BDL model. All animals in the groups were sacrificed at the end of treatment, and their sera and liver tissues were collected.

For CCl_4_-induced liver fibrosis mice model, Mice were randomly divided into 5 groups: (1) control; (2) CCl_4_; (3) CCl_4_ +5 mg/kg LIG, (4) CCl_4_ +10 mg/kg LIG, (5) CCl_4_ +20 mg/kg LIG, n = 8. CCl_4_ is diluted with corn oil. For group (2-5), mice were orally administered with CCl_4_ at a dose of 1.6 mg/kg twice a week for 8 weeks. For group (3-5), different dose of LIG was orally administrated once daily. All animals in the groups were sacrificed at the end of treatment, and their sera and liver tissues were collected.

Tmem173^-/-^ mice (STING knockout) were purchased from Shanghai Model Organisms Center, Inc. (Shanghai, China). Tmem173^-/-^mice were housed under the same condition as mentioned above. After that, mice were randomly divided into 5 group: (1) sham, (2) BDL, (3) BDL + 5 mg/kg LIG, (4) BDL + 10 mg/kg LIG and (5) BDL + 20 mg/kg LIG, n = 5. Firstly, mice were pre-administrated with various doses of LIG for 2 days (i.g.). After 2 days of surgery, mice were subjected to BDL surgery and treated with LIG (i.g.) for another 4 days. After treatment and anesthesia, mice were collected with sera and livers.

In order to especially knockout STING in liver, we applied AAV8-EGFP-shSTING adeno-associated virus to build the mice model. The mice were housed under the same condition. Then, mice were divided into sham + AAV8-control-EGFP-shRNA, BDL + AAV8-control-EGFP-shRNA, BDL + AAV8-control-EGFP-shRNA + 20 mg/kg LIG, BDL + AAV8-EGFP-shSTING and BDL + AAV8-EGFP-shSTING + 20 mg/kg LIG, n = 5. Firstly, mice at 8 weeks of age were injected from the tail vein with AAV-control-shRNA (1 × 10^11^ v.g), and AAV8-EGFP-shSTING (1 × 10^11^, v.g). After 14 days, mice were pre-administered with various doses of LIG (i.g.) for 2 days before undergoing BDL surgery. After 2 days recovery, mice received different doses of LIG (i.g.) for successive 4 days. After treatment and anesthesia, the sera and liver tissues of mice were collected.

### RNA-sequencing (RNA-seq) analysis and bioinformatics analysis

Firstly, RNA from sham, BDL, BDL + LIG (10 mg/kg) group mice tissue or control, 5 μg/mL TGF-β, 5 μg/mL TGF-β + 20 μM LIG group LX-2 cells were extracted by TRIZOL regent. After cDNA synthesized, the library fragments were purified to enrich 250-300 bp cDNA fragments preferentially. Then the sequencing library was performed through an Illumina Novaseq platform to get gene expression data normalization and differential gene expression. Furthermore, differentially expressed genes (DEGs) were analyzed by gene ontology (GO) enrichment analysis and kyoto encyclopedia of genes and genomes (KEGG) enrichment analysis by using cluster profiler R package. Based on the GO and KEGG datasets, gene set enrichment analysis (GSEA) was used to further analyze the differential expression data. Moreover, the analysis and visualization of heat maps, cellular characteristic analysis, and cellular correlation analysis were all conducted under the R 4.3.1 version.

### Cell senescence flow cytometry

The hepatocyte senescence and HSC senescence was detected by using the cell event™ senescence green flow cytometry assay kit (C10840, Thermo Fisher). First, cell samples from different groups were digested with trypsin enzyme and then washed with PBS. After centrifugation, the cells were resuspended in PBS to a concentration of 0.5 × 10^6^-1 × 10^6^ cells per 100 μL. After fixing with paraformaldehyde, the work dyeing liquid of cells that according to the kit instruction configured in advance dyed cells in the incubation box without carbon dioxide at 37 ℃ for 2 h. The hepatocyte senescence and HSC senescence analysis was performed using CytoFLEX at 488 nm wavelength.

### G-actin polymerization assay

The effect of LIG on interrupting G-actin polymerization was detected by the actin polymerization biochem kit™ muscle actin (BK003, Cytoskeleton, Denver, CO, USA). Specifically, according to the instruction, two wells of 96-well plate (A1, B1) were added with 200 μL G-Buffer, while another 6 wells (C1-H1) were added with 200 μL G-actin stock. Then the plate was put into the fluorometer for 3 minutes. Ather that, E1 and F1 wells were added with 20 μL DMSO, G1 and H1 wells were added with 20 μL LIG (20 μM). The reading was continued for 20 min after 5 s shaking. Finally, the eight wells were added with 20 μL of 10 x actin polymerization buffer. The readings were continued for 1 h until the readings were stable.

### Nuclear membrane permeability assay

FITC-Dextran of different molecular weights exhibits distinct nuclear envelope permeability. The FITC-Dextran with different molecular weights were prepared into solutions with ddH_2_O according to the instructions. Following a 2-hour co-incubation of live cells with 10 kD, 20 kD and 70 kD FITC-dextran probes, the live cells were washed twice to move uninternalized probes and stained with DAPI, enabling that all detectable fluorescence signals originated from FITC-dextran probes had entered the cells. Then the cells were imaged by Olympus FV3000 confocal laser scanning microscopy (Tokyo, Japan).

### *In vitro* G-actin/ F-actin ratio assay

G-actin and F-actin were isolated by using the F-actin assay kit (BK037, Cytoskeleton, Denver, CO, USA). Specifically, after scraping the cells in LAS2 buffer, the cells were centrifuged at 350 × g for 5 min. Then the supernatant was isolated, which was centrifuged at 1000,000 × g for 1 h by using the ultracentrifuge. The supernatant was G-actin, while F-actin was in the precipitate. After separation of G-actin, F-actin was depolymerized by using F-actin depolymerization agent. Thereafter, the expression of G-actin and F-actin in different groups was detected by western blot (WB).

### Primary hepatic cell isolation

HSCs and hepatocytes were extracted from mouse liver in sham, BDL and BDL + LIG (10 mg/kg) group. Briefly, after removing blood in livers by perfusion, livers were digested by William's E solutions containing collagenase (C5138, Sigma,). The digested tissues were first filtered through a 100 μm mesh, and then centrifuged to separate the supernatant, which contains non-parenchymal cells, from the pellet, which contains hepatocytes. For the precipitate cells, after washing with complete growth medium of William's E, hepatocytes were obtained. The supernatant was subjected to centrifugation at first. Then the precipitation was obtained washed with DMEM containing 1% PS three times. Then, the gradient column of percoll (P4937, Sigma) was formed by adding different concentrations of percoll to obtain HSCs.

### Plasmid transfection

The LX-2 cells were seeded in 6-well plates to achieve a cell density of 70%-80% at the time of transfection. First, after replacing the cell culture medium with opti-MEM medium, 2.5 μg hYAP1 overexpression plasmid or control plasmid were mixed with lipofectamine 3000 reagent (L3000015, Invitrogen, USA) and added into wells. After 6-8 h of transfection, western blot and quantitative real-time RT-PCR were performed for verification.

### Immunoprecipitation (IP)

IP analysis was used to detect the physical interaction between Lamin A/C and actin, utilizing protein A/G beads for antibody-antigen complex isolation during the assay. Briefly, Lamin A/C antibody was combined with protein A/G beads (sc-2003, Santa Cruz, CA). Then the protein samples were collected by lysis buffer and mixed with the Lamin A/C -protein A/G beads. Finally, the bound samples were analyzed by western blot.

### Statistical analysis

All experimental data were replicated a minimum of three times and analyzed using SPSS or GraphPad Prism software. Results are presented as mean ± SD, with group differences assessed through one-way analysis of variance (ANOVA). A *P*-value of less than 0.05 was deemed statistically significant. Additional methods are detailed in the supplementary document.

## Results

### LIG ameliorates experimental liver fibrosis and regulates HSC functions

**Figure 1:**
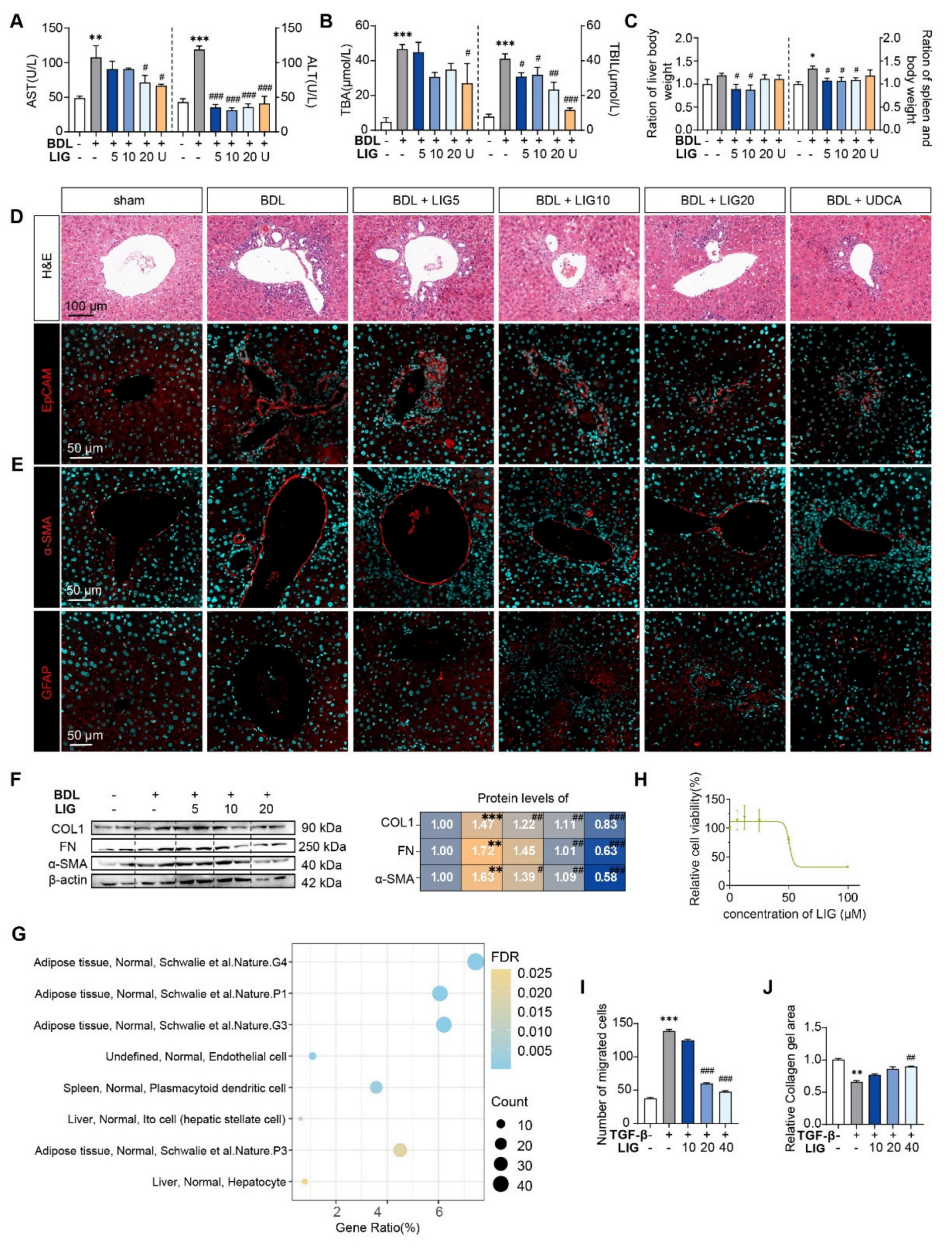
** LIG ameliorates experimental liver fibrosis and regulates HSC functions.** (**A**) Serum levels of ALT and AST. (**B**) Serum levels of TBA and TBIL. (**C**) Ratios of liver or spleen weight to body weight. (**D**) Representative images of H&E staining and IF staining for EpCAM. (**E**) IF staining for α-SMA and GFAP. (**F**) Protein levels of COL1, FN, and α-SMA were evaluated by WB and normalized to β-actin. (**G**) Cell-type enrichment analysis of DEGs in the BDL + LIG group vs the BDL group. (**H**) The IC_50_ assay of LIG in LX-2 cells. (**I**) The quantification result of transwell migration assay. (**J**) The quantification result of collagen gel. Statistical significance: *P < 0.05, **P < 0.01, *** P < 0.001, compared with the sham group; ^#^* P* < 0.05, ^##^* P* < 0.01, ^###^* P* < 0.001, compared with the BDL group.

To evaluate whether LIG produces the anti-fibrotic effects in the liver, we established BDL- and CCl_4_ -induced liver fibrosis mice model and administered LIG at various oral doses (**Figure S1A** and **S2A**). BDL surgery resulted in significant elevations in the serum levels of aspartate aminotransferase (AST), alanine aminotransferase (ALT), total bile acid (TBA) and total bilirubin (TBIL), which were reduced with LIG treatment. High doses of LIG (20 mg/kg) showed similar improvements compared with the positive control drug, UDCA (**Figure 1A**-**B**). With CCl_4_ administration, similar trends for serum levels of ALT and AST were observed (**Figure S1B**). Additionally, BDL surgery caused significant increases in liver and spleen weights, which were reduced by LIG, indicating amelioration of swollen liver and severe inflammatory stress (**Figure 1C**). Consistently, histopathological and IF staining showed that LIG mitigated BDL- and CCl_4_-induced inflammatory infiltration, structural disorders, collagen deposition in liver (**Figure 1D, S1C** and **S2B-C**). Importantly, a critical event in liver fibrosis is the transdifferentiation of quiescent HSCs into myofibroblasts. Glial fibrillary acidic protein (Gfap) and lecithin retinol acyltransferase (Lrat) are the marker of quiescent HSCs, while α-smooth muscle actin (α-SMA) is a marker of activated HSCs. IF staining, qPCR and WB results indicated that the BDL surgery and CCl_4_ administration increased the number of activated HSCs and decreased the number of quiescent HSCs (**Figure 1E, S1D** and **S2D-G**). These changes were effectively reversed by LIG treatment. RNA-seq analysis of liver samples from sham, BDL and BDL + LIG (10 mg/kg) group showed that the expression of anti-fibrosis genes including Sad1 and UNC84 domain containing 2 (*Sun2*) and lysyl oxidase like 3 (*Loxl3*) 23, 24, was reduced in BDL group but increased in LIG group. Conversely, pro-fibrotic genes such as smooth muscle alpha actin (*Acta2*), TIMP metallopeptidase inhibitor 1 (*Timp1*) and fibronectin 1 (*Fn1*) were elevated in the BDL group and decreased in the LIG group (**Figure S3A**). Then, WB and qPCR analyses further confirmed the LIG ameliorated BDL- and CCl_4_-induced liver fibrosis (**Figure 1F, S1E-F** and** S3B**). To investigate the anti-fibrotic mechanisms of LIG, in **Figure S3C**, we performed transcriptomic analysis and identified 609 differentially expressed genes (DEGs) between the bile duct ligation (BDL) and sham groups, 2288 DEGs between the BDL + LIG and BDL groups (criteria: *P* < 0.05 and |log₂ (fold change) | > 1). The raw RNA-seq data in our analyses have been uploaded to Gene Expression Omnibus database (https://www.ncbi.nlm.nih.gov/geo) under accession number GSE299743. Next, we applied cell-type enrichment analysis to forecast the specific hepatic cell types targeted by LIG and found that, HSC were the possible target cells of LIG (**Figure 1G**). After detecting the IC_50_ of LIG in human HSCs, LX-2 cells (**Figure 1H**), we found that LIG could inhibit the activation of LX-2 cells (**Figure S3D-E**). Also, LIG inhibit the migration of LX-2 cells (**Figure 1I** and** S3F**). Moreover, under the treatment of TGF-β, the original gel area shrank significantly due to a potent contraction force. However, LIG exerted a remarkable effect of enlarging the original gel area and enhancing the resistance to collagen contraction to the maximum extent, as indicated by** Figure 1J** and** S3G**.

### LIG induces HSC microfilament rearrangement through binding with G-actin

**Figure 2:**
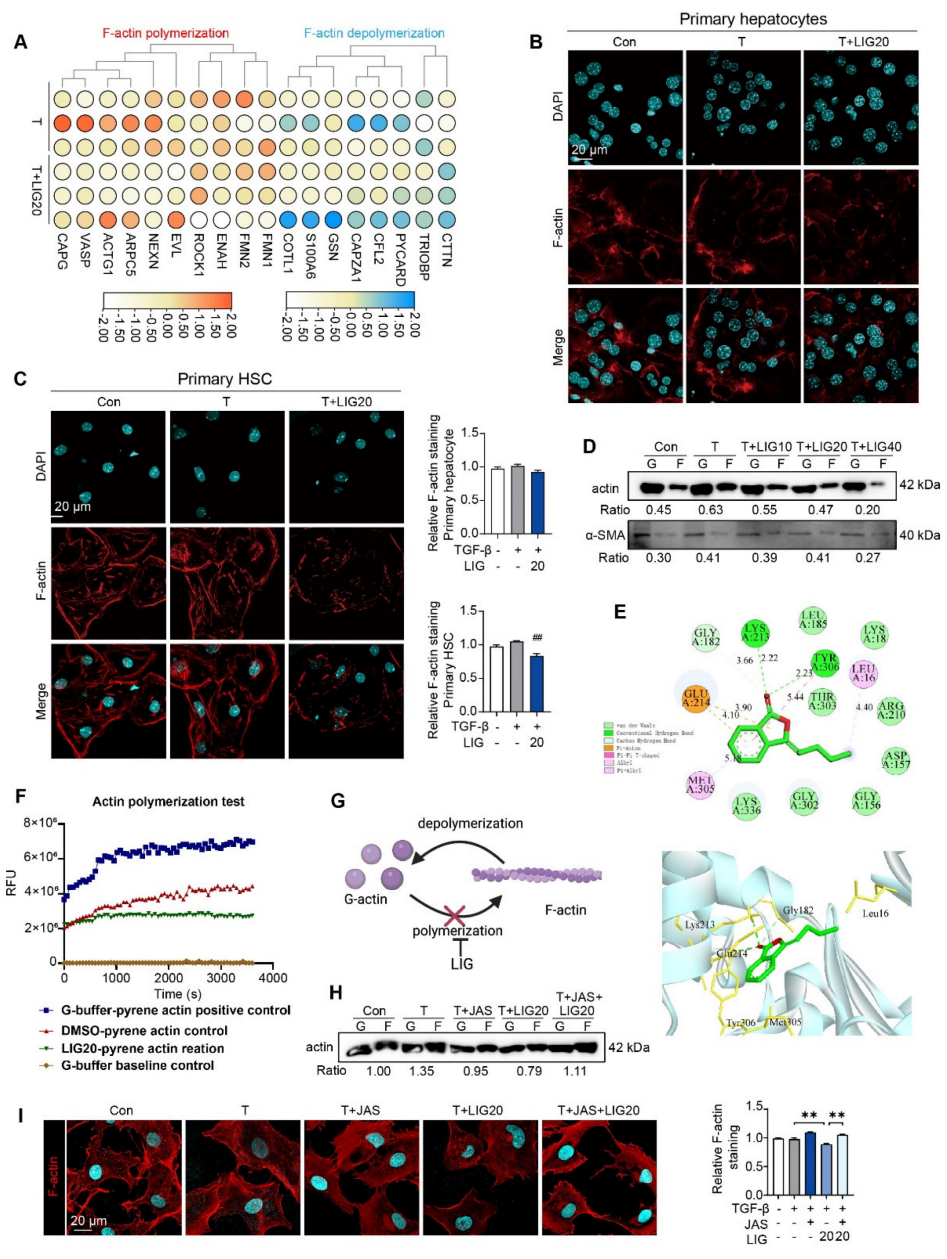
** LIG induces HSC microfilament rearrangement.** (**A**) The heatmap of F-actin polymerization and depolymerization genes. (**B-C**) IF staining of F-actin in primary hepatocyte (**B**) and HSC (**C**), accompanied by the quantification results. (**D**) F-actin and G-actin extracted from LX-2 cells treated with TGF-β or LIG for 24 h are subjected to western blot with actin and α-SMA. (**E**) The diagram of LIG and G-actin molecular docking. (**F**) G-action polymerization assay by actin polymerization kit. (**G**) The diagram illustrates how LIG inhibits G-actin polymerization. (**H**) F-actin and G-actin that extracted from LX-2 cells treated with TGF-β, JAS or LIG for 24 h are isolated and detected with WB. (**I**) IF co-staining of F-actin (red) and DAPI (cyan). Statistical significance: ^##^* P* < 0.01, compared with the TGF-β group; **P < 0.01.

We applied RNA-seq to analyzing the function of LIG *in vitro*. 153 DEGs were identified between TGF-β group and LIG group (*P* < 0.05 and a |log foldchange (FC)| > 1) (**Figure S4A**). The raw RNA-seq data in our analyses have been uploaded to Gene Expression Omnibus database (https://www.ncbi.nlm.nih.gov/geo) under accession number GSE299450. It has been reported that in the context of liver fibrosis, actin is predominantly present in the form of F-actin in HSCs 25. The heatmap showed that F-actin depolymerization genes were upregulated while F-actin polymerization genes were downregulated with LIG treatment (**Figure 2A**). The *in vitro* IF staining of F-actin in primary HSCs and hepatocytes indicated that LIG specifically inhibited the F-actin in HSCs (**Figure 2B-C**). Moreover, the co-staining of F-actin and α-SMA in LX-2 demonstrated that LIG inhibited the incorporation of α-SMA with F-actin (**Figure S4B**). Similarly, Also, the G-actin/ F-actin ratio assay showed LIG significantly suppressed G-actin polymerization in LX-2 cells (**Figure 2D**). We also applied another HSC cell line, HSC-T6 cells and found the similar anti-fibrotic effects and disruption of microfilaments with LIG treatment (**Figure S5A-C**). To deeply investigate the mechanism of LIG, we first applied molecular docking and found that LIG could directly combine with G-actin (**Figure 4E**). The results of the* in vitro* experimental actin polymerization assay indicated that LIG directly prevented the polymerization of G-actin (**Figure 4F**), leading to a reduction in F-actin levels (**Figure 4G**). In addition, the actin-stabilizing agent, JAS was used to further assess the effects of LIG on microfilament stability. The application of JAS reversed the microfilament depolymerization effects of LIG in LX-2 cells (**Figure 2H-I** and **Figure S4C**).

### The anti-fibrotic effects of LIG are related with cellular senescence

**Figure 3:**
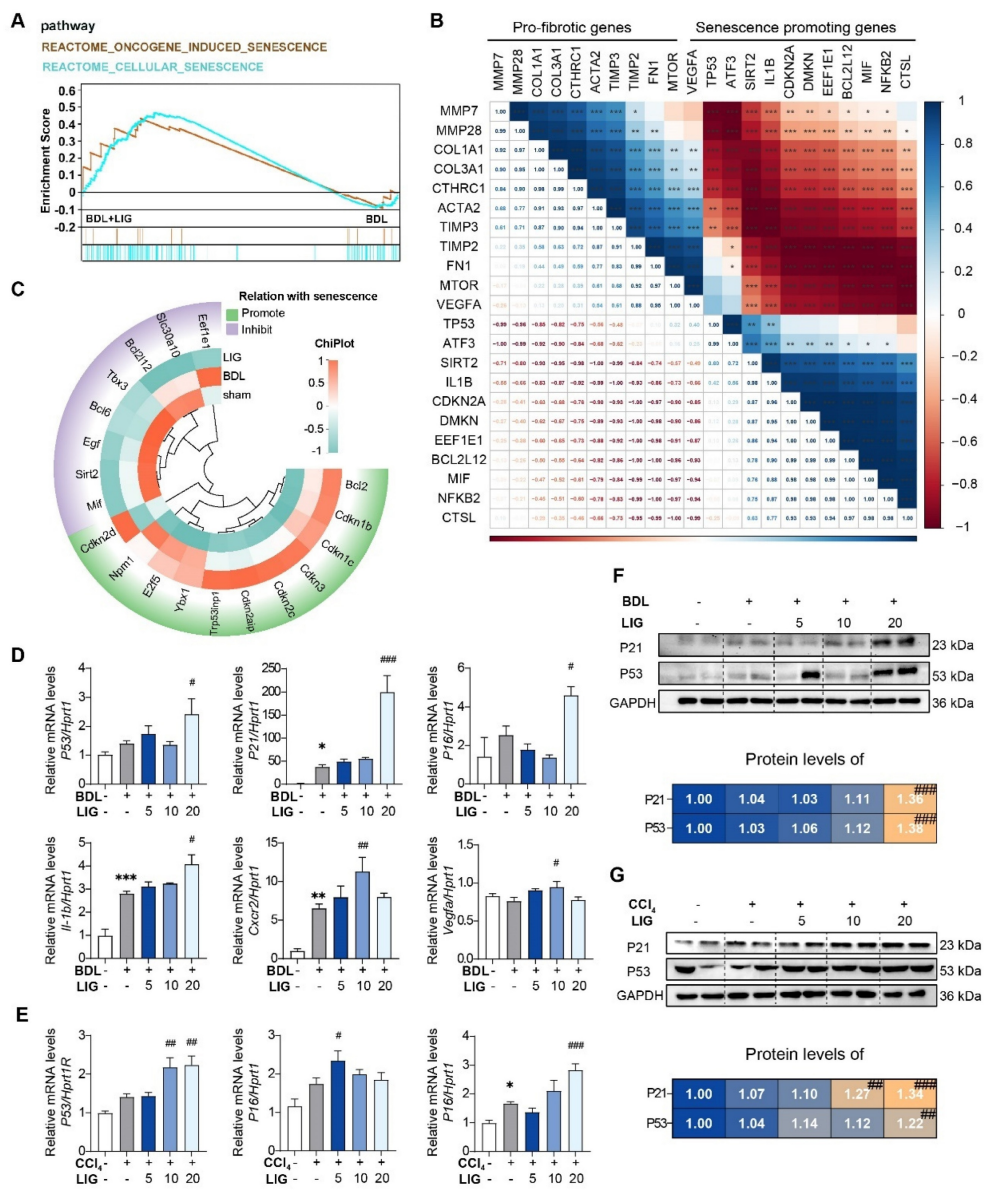
** The anti-fibrotic effects of LIG are related with cellular senescence.** (**A**) GSEA analysis of senescence-related pathway. (**B**) The correlation of pro-fibrotic genes and senescence promoting genes. (**C**) The heatmap of senescence-related marker genes. (**D-E**) Relative mRNA levels of cell cycle-related genes and SASPs are determined by qPCR and normalized with *Hprt1* in BDL-(**D**) and CCl_4_-(**E**) induced fibrosis model. (**F-G**) The protein levels of P21 and P53 in BDL-(**F**) and CCl_4_-(**G**)induced fibrosis model are determined by WB and normalized by GAPDH. Statistical significance: * P < 0.05, ** P < 0.01, *** P < 0.001, compared with sham/con group; *^#^ P* < 0.05, *^##^ P* < 0.01, *^###^ P* < 0.001, compared with the BDL/CCl_4_ group.

The RNA-seq results of mice showed that the top 20 GO analysis results indicated that the DEGs between the BDL + LIG and BDL groups were enriched in “cell aging”, “negative regulation of cellular senescence” (**Figure S6A**). DEGs between the BDL + LIG and BDL were enriched in “Cellular senescence” and “p53 signaling pathway” in KEGG analysis, indicating the importance of cellular senescence in the anti-fibrotic effects of LIG (**Figure S6B**). GSEA analysis confirmed that senescence-related pathways were positively correlated with LIG treatment (**Figure 3A**). To further study whether the induction of cellular senescence was related to liver fibrosis process, a correlation analysis was performed between fibrosis-related genes and senescence-related genes.

As shown in (**Figure 3B**), the pro-fibrotic genes were negatively correlated with senescence-promoting genes, indicating cellular senescence might conduct the liver fibrosis process. The heatmap showed that senescence promoting genes were markedly upregulated in the BDL + LIG group compared with the BDL group, while the genes that prevent senescence process were downregulated (**Figure 3C**). The cell cycle-related genes such as tumor protein p53 (*P53*), cyclin dependent kinase inhibitor 1A (*P21*), cyclin dependent kinase inhibitor 2A (*P16*), as well as SASPs including interleukin 1 beta (*Il-1b*), C-X-C motif chemokine receptor 2 (*Cxcr2*) and vascular endothelial growth factor A (*Vegfa*) were significantly upregulated with the administration of high dose of LIG in BDL- and CCl_4_-induced fibrosis model (**Figure 3D-E**). The WB results consistently supported that the P21 and P53 were increased with LIG treatment (**Figure 3F-G**) Based on these results, effects of LIG treatment alone were evaluated *in vivo*. The results demonstrated that LIG treatment alone had no significant effects on serum biochemical parameters (**Figure S7A**), pathological results (**Figure S7B**), fibrosis-related markers (**Figure S7C**), and senescence-associated genes (**Figure S7D**).

### LIG targeting of HSC senescence in the liver is linked to microfilament remodeling

**Figure 4:**
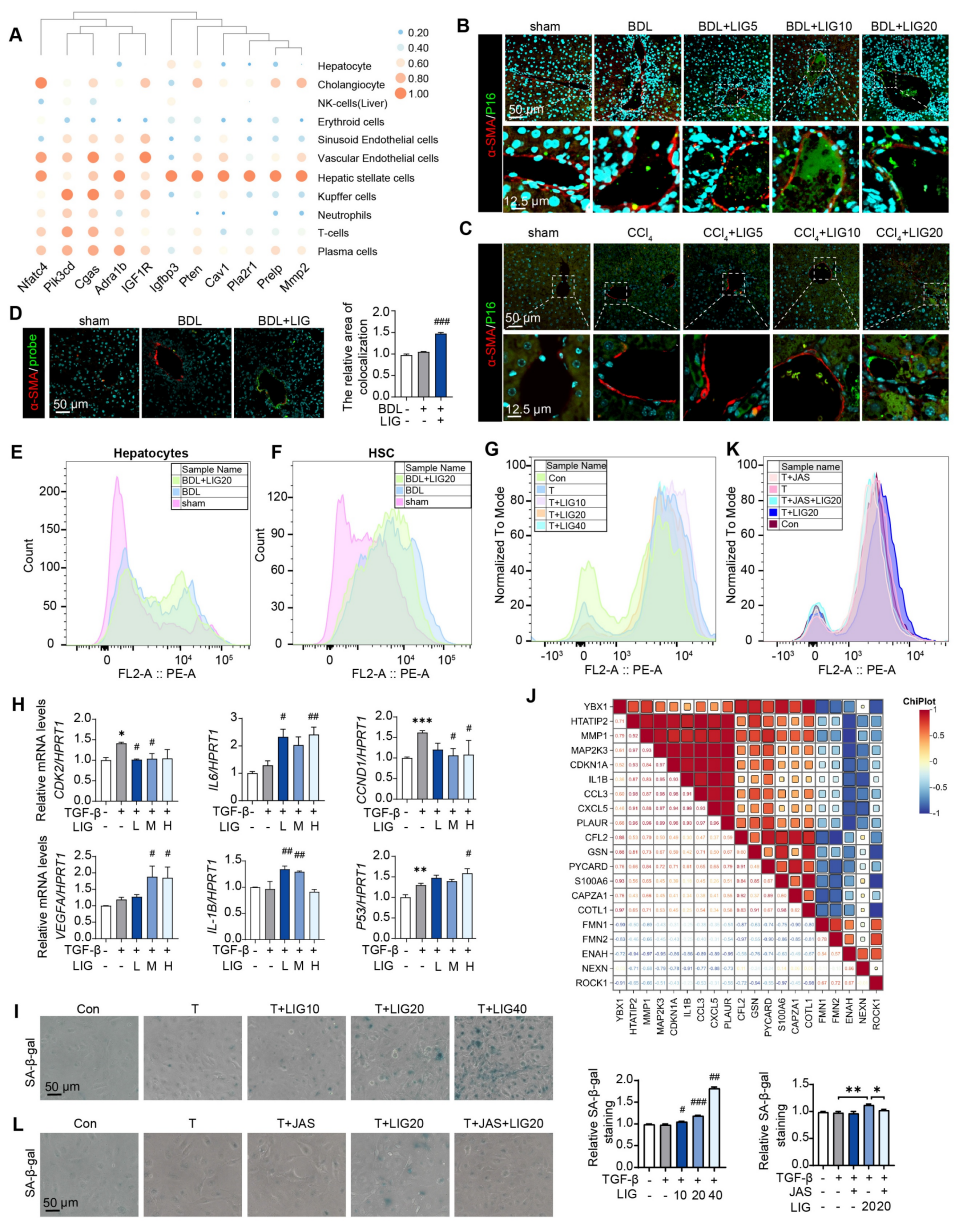
** LIG targeting of HSC senescence in the liver is linked to microfilament remodeling.** (**A**) Predicted correlations of DEGs in the BDL + LIG group vs the BDL group with different liver cell subtypes. (**B-C**) Representative images of the co-staining of α-SMA and P16 in BDL (**B**)- and CCl4 (**C**)-induced fibrosis. The quantification of the immunostaining was performed using Image J software. (**D**) Co-staining of α-SMA (red) and senescence probe (green). (**E-G**) The primary mouse hepatocyte (**E**), primary mouse HSC (**F**) and LX-2 cell (**G**) senescence analysis by flow cytometry. (**H**) Relative mRNA levels of *CCND1*, *CDK2*, *P53*, *IL-6*, *VEGFA* and *IL-1B* in LX-2 cells by qPCR and normalized with *HPRT1*. (**I**) SA-β-gal staining in LX-2 cells. (**J**) The correlation of senescence-related marker genes and cytoskeleton-related marker genes. (**K**) The LX-2 cells senescence analysis with JAS by flow cytometry. (**L**) SA-β-gal staining of LX-2 cells with JAS. Statistical significance: * P < 0.05, ** P < 0.01, *** P < 0.001, compared with sham group; *^#^ P* < 0.05, *^##^ P* < 0.01, *^###^ P* < 0.001, compared with the TGF-β group.

According to the predicted correlations, the cellular senescence DEGs were markedly related to HSCs (**Figure 4A**). Moreover, in BDL-induced liver fibrosis model, the cellular senescence marker P16 were co-stained with the HSC maker, α-SMA (**Figure 4B** and **Figure S8A**), hepatocyte marker, ALB (**Figure S8B**) and the liver sinusoidal endothelial cell marker, LYVE1 (**Figure S8C**). Similar results were shown in CCl_4_ mice model (**Figure 4C** and **Figure S9A**). Also, we applied senescence probe and found LIG significantly induced HSC senescence (**Figure 4D** and **Figure S9B)**. As HSCs showed senescence phenotype, we extracted the primary HSCs and hepatocytes from the sham, BDL and BDL + LIG group mice and performed cell senescence flow cytometry. Results indicated an increase in senescent primary HSCs in the BDL + LIG group, while senescent primary hepatocyte numbers remained unchanged (**Figure 4E-F**). Also, the GO analysis of RNA-seq results of LX-2 showed that DEGs were enriched not only in collagen-related process, but also “aging” process (**Figure S10A**). GSEA also indicated that senescence-related pathways were positively correlated with LIG treatment (**Figure S10B**). Thus, cell senescence flow cytometry and qPCR results showed that LIG could induce cellular senescence *in vitro* (**Figure 4G**-**H**). Additionally, the results of senescence-associated beta-galactosidase (SA-β-gal) staining for detecting senescent cells and EdU staining for evaluating proliferative capacity indicated that LIG treatment accelerated LX-2 cell senescence (**Figure 4I** and **Figure S10C**). Annexin V/PI assay demonstrated no LIG-induced apoptosis in HSCs (**Figure S10D**). We also found the similar senescence induction of LIG treatment in HSC-T6 cells (**Figure S11A-C**). These results suggested that among the cells regulated by LIG, cellular senescence predominantly occurs in HSCs. Interestingly, we found that the senescence promoting genes were positively correlated with F-actin depolymerization genes and negatively correlated with F-actin polymerization genes (**Figure 4J**). Furthermore, SA-β-gal staining, cell senescence flow cytometry and EdU staining consistently indicated that the pro-senescence effects of LIG were also reversed by JAS (**Figure 4K-L** and **Figure S12A**).

### Cytoskeletal remodeling induced by LIG inhibits the translocation of YAP from the cytoplasm to the nucleus in HSCs

**Figure 5:**
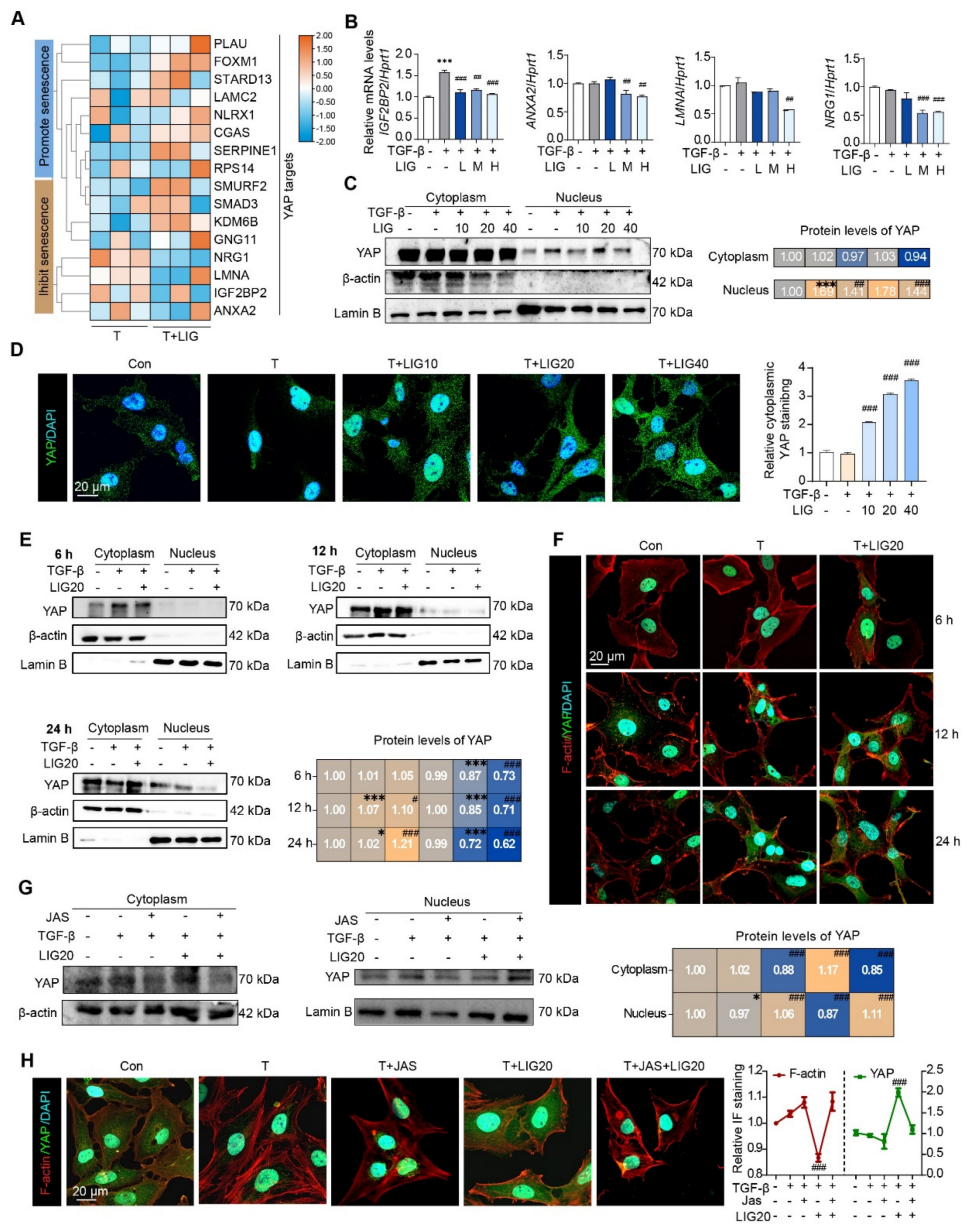
** Cytoskeletal remodeling induced by ligustilide inhibits the translocation of YAP from the cytoplasm to the nucleus in HSCs.** (**A**) The heatmap of YAP targes genes associated with senescence. (**B**) Relative mRNA levels of *IGFBP2*, *ANXA2*, *LMNA* and *NRGF1* by qPCR and normalized with *HPRT1*. (**C**) WB analysis to detect the expression of YAP, actin and lamin B for the nucleus and cytoplasmic proteins extracted from LX-2 cells with TGF-β or LIG treatment. (**D**) Immunofluorescence for YAP (green) and DAPI (cyan) merge images. (**E**) The protein levels of YAP were detected by WB extracted from LX-2 cells treated with TGF-β, LIG or JAS at 6 h, 12 h, or 24 h. (**F**) Immunofluorescence for YAP (green), F-actin (red) and DAPI (cyan) merge images treated with TGF-β or LIG at 6 h, 12 h or 24 h. (**G**) The protein levels of YAP were determined by western blot analysis and normalized by β-actin after treating with JAS. (**H**) Immunofluorescence for YAP (green), F-actin (red) and DAPI (cyan) merge images after treating with JAS. Statistical significance: * P < 0.05, ** P < 0.01, *** P < 0.001, compared with con group in nuclues or cytoplasm; *^#^ P* < 0.05, *^##^ P* < 0.01, *^###^ P* < 0.001, compared with the TGF-β group in nuclues or cytoplasm.

Notably, as determined from RNA-seq data of HSCs and mouse liver, several genes that either promote or inhibit HSC senescence, were also identified as YAP target genes (**Figure 5A** and **Figure S13A**). In addition, the expression of p-YAP that is unable to enter the nucleus was upregulated in LX-2 cells (**Figure S13B**). Especially, the expression of the downstream genes of YAP including insulin like growth factor 2 mRNA binding protein 2 (*IGF2BP2*), annexin A2 (*ANXA2*), lamin A/C (*LMNA*) and neuregulin 1 (*NRG1*) was significantly downregulated with LIG treatment (**Figure 5B**). Then, we isolated protein from HSC cytoplasm and nucleus and detected the expression of YAP. As shown in** (Figure 5C)**, the protein level of YAP in the nucleus was upregulated, but this effect was decreased with LIG treatment. The IF staining also showed that YAP was mainly located in the nucleus in the TGF-β group, whereas it translocated to cytoplasm with the presence of LIG in both LX-2 cells and primary HSCs (**Figure 5D** and **Figure S13C-D**). As reported, YAP functions as a nuclear transducer of ECM mechanical signals, and this regulatory process depends on the dynamic F-actin network 15. We assessed the levels of F-actin and the nuclear-cytoplasmic distribution of YAP under the same LIG treatment duration. After 12 h of LIG treatment, F-actin depolymerization and YAP nuclear translocation occurred simultaneously (**Figure 5E-F** and **Figure S13E**). After 24 h of treatment, further F-actin depolymerization was accompanied by extensive YAP nuclear translocation. To further verify the conduction of F-actin on YAP translocation, we applied JAS to stabilize F-actin and found that, with the protection of JAS on the depolymerization of F-actin, YAP located in nucleus was increased. Furthermore, the reduction of YAP localization in the nucleus induced by LIG treatment was reversed by JAS (**Figure 5G-H** and **Figure S13F**).

### Lamin A/C, a downstream factor of YAP, is implicated in LIG-induced senescence of HSCs

**Figure 6:**
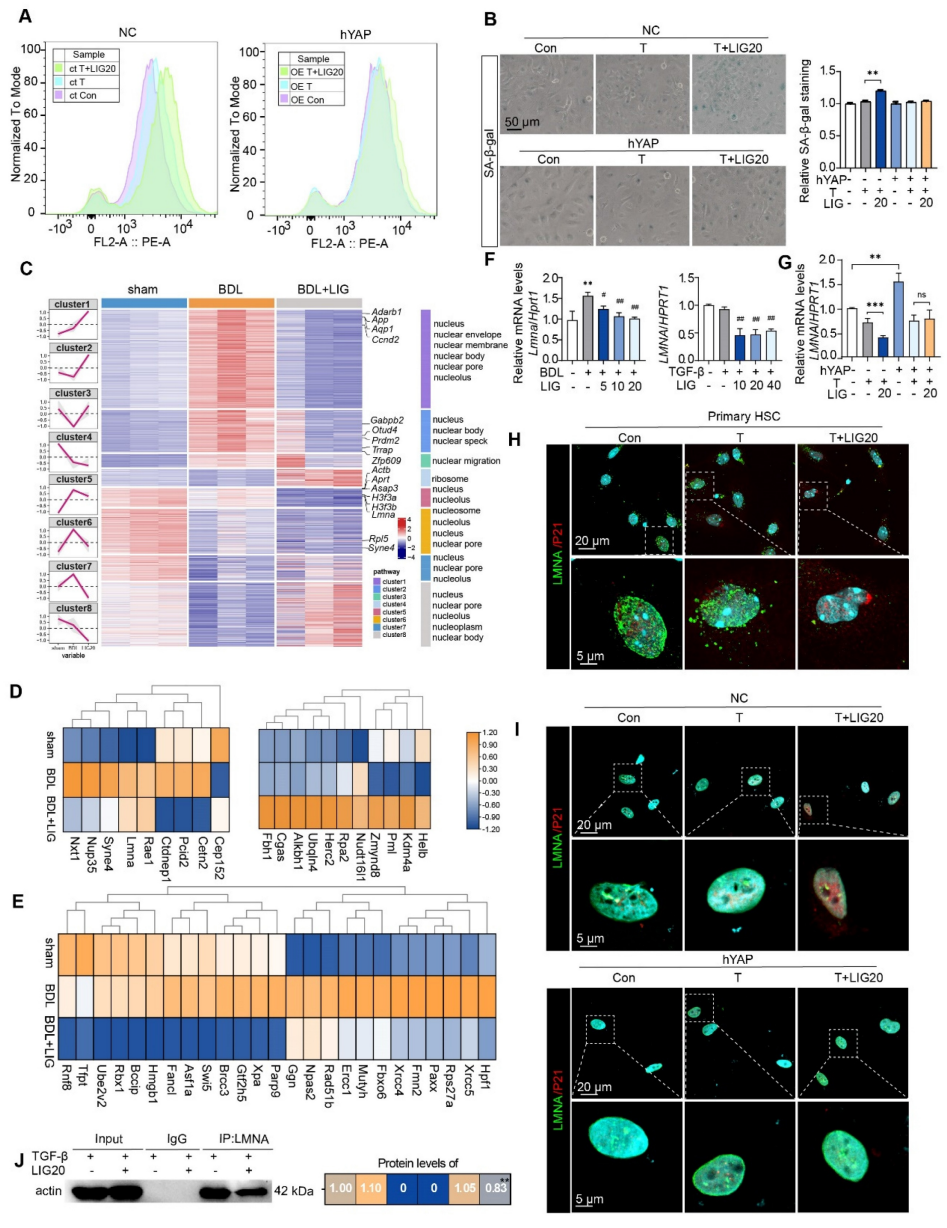
** LMNA participates in ligustilide induced HSC senescence.** (**A**) LX-2 cell senescence is assessed using flow cytometry following ct or OE-hYAP plasmid transfection. (**B**) SA-β-gal staining of LX-2 cells following ct or OE-hYAP plasmid transfection, accompanied by the quantification results. (**C**) Combined diagram of heatmap, GO functional annotation and clusters. (**D**) The heatmaps of nuclear membrane and nuclear pores related genes. (**E**) The heatmaps of genes related with DNA damage and repair. (**F-G**) Relative mRNA levels of *LMNA* in mouse liver, LX-2 cells (**F**), and LX-2 cells treated with ct or OE-hYAP plasmid transfection (**G**) are measured by qPCR and normalized to *Hprt1*. (**H**) IF co-staining of LMNA (green), P21 (red) and DAPI (cyan) in primary HSCs. (**I**) IF co-staining of LMNA (green), P21 (red) and DAPI (cyan) following ct or OE-hYAP plasmid transfection. (**J**) IP analysis of the combination of LMNA and F-actin. Statistical significance: * P < 0.05, ** P < 0.01, *** P < 0.001.

Firstly, LX-2 cells were transfected with hYAP plasmids to overexpress YAP. When YAP was overexpressed (**Figure S14A**), cell senescence flow cytometry (**Figure 6A**), SA-β-gal staining (**Figure 6B**) and EdU staining (**Figure S14B**) results consistently indicated that the HSC senescence induced by LIG was reduced. Moreover, the increased expression of senescence-promoting genes and decreased expression of pro-fibrotic genes induced by LIG were reversed (**Figure S14C**). The ClusterGVis package was applied to further analyze RNA-seq data through which, we created a combined visualization of trend plots, heatmaps and GO functional annotation. Interestingly, we found several clusters enriched in nucleus-related processes (**Figure 6C**). After analyzing the nuclear envelope integrity-maintaining genes, we found the genes that destroy the integrity of nuclear envelope were upregulated with LIG treatment, while the genes that protect the integrity of nuclear envelope were downregulated (**Figure 6D**). The heatmap of DNA damage and repair genes indicated that the genes promoting DNA damage were upregulated in LIG group, while the genes conducting DNA repair were downregulated (**Figure 6E**). Notably, LMNA, a key component of the nuclear envelope that belongs to the cytoskeleton component, intermediate filament protein family 26, not only showed in the **Figure 6D**, but also was a downstream target of YAP (**Figure 5A**). Thus, we suspended the downregulation of LMNA was corresponded with the transcription factor YAP. LMNA mRNA expression was significantly reduced in the TGF-β + LIG group compared to the TGF-β group in HSCs, and in BDL + LIG-treated mice compared to the BDL group (**Figure 6F**), which was consistent with the RNA-seq results in **Figure 6E**. In addition, the overexpression of YAP upregulated the mRNA expression of LMNA, effectively reversing the LIG-induced dysregulation of LMNA (**Figure 6G**). Consistently, in conjunction with the upregulation of P21, LMNA levels were significantly diminished in the TGF-β + LIG (T + LIG) group in primary HSCs (**Figure 6H** and **Figure S14D**). Also, upon overexpression of YAP, the structure of perinuclear LMNA remained intact with LIG treatment, coinciding with low expression levels of P21 (**Figure 6I** and **Figure S14E**). As intermediate filaments normally connect with F-actin to maintain nuclear integrity 27, we applied IP experiment and found that the interaction between Lamin A/C and F-actin was reduced after treatment with LIG, indicating the possibility that nucleus might be broken in this case (**Figure 6J**).

### The dsDNA-cGAS-STING pathway is responsible for mediating the senescence effects of LIG on HSCs

**Figure 7:**
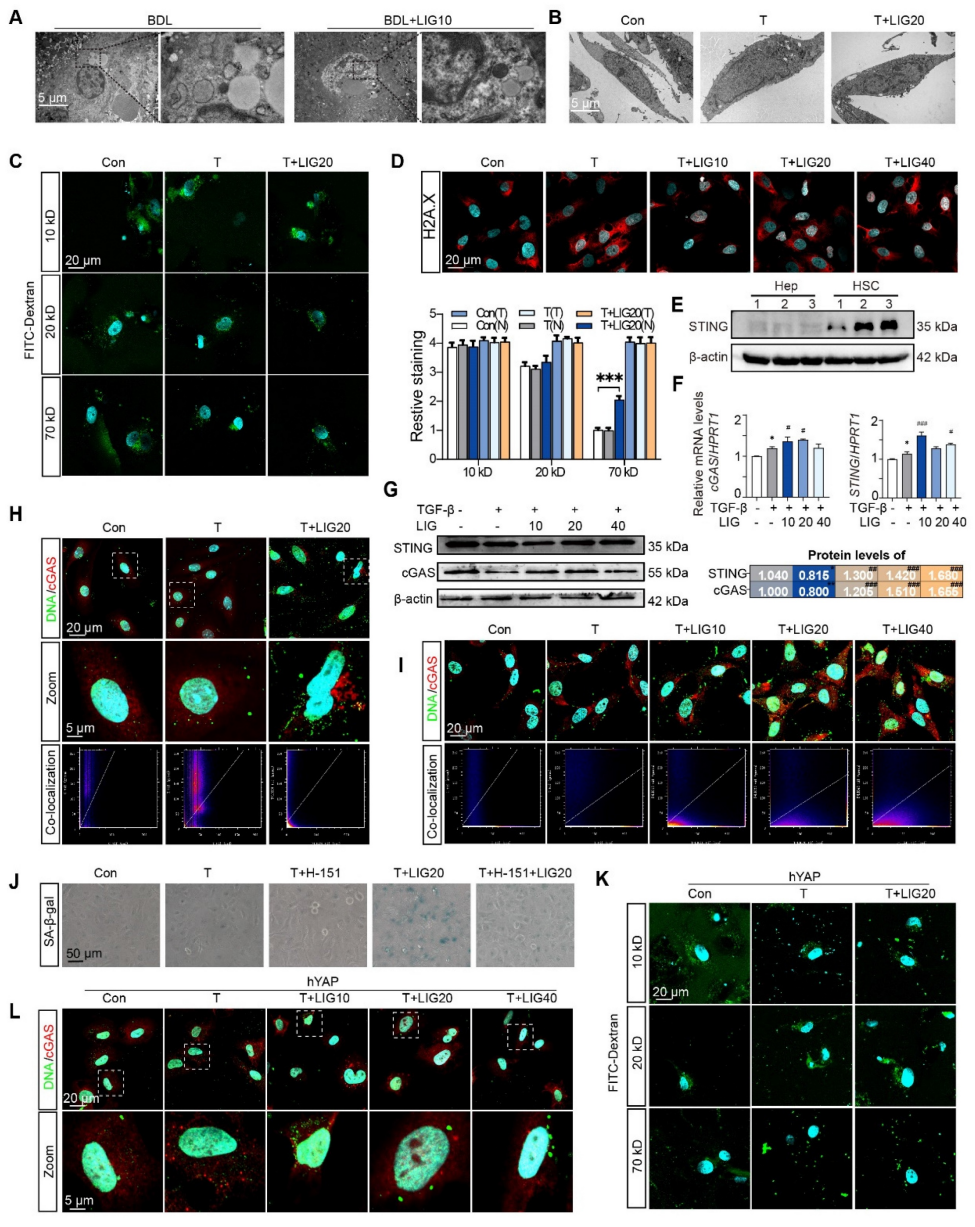
** The dsDNA-cGAS-STING is responsible for the senescence effects of ligustilide on HSCs.** (**A**-**B**) Scanning electron microscope picture in liver (**A**) and LX-2 cells (**B**). (**C**) IF co-staining of 10 kD, 20 kD or 70 kD FITC-Dextran (green) and DAPI (cyan), accompanied by the quantification of the total IF siginalings (T) and nuclear IF siginalings (N). (**D**) IF co-staining of γH2AX (red) and DAPI (cyan) in LX-2 cells. (**E**) The protein expression of STING in primary hepatocytes and HSCs from 3 mice. (**F**) Relative mRNA levels of *cGAS* and *STING* in LX-2 cells were determined by qPCR and normalized with *Hprt1*. (**G**) The protein levels of cGAS and STING in LX-2 cells were determined by WB and normalized by β-actin. (**H-I**) IF co-staining of cGAS (red) and α-SMA (green) in primary HSCs (**H**) and LX-2 cells (**I**). (**J**) SA-β-gal staining of LX-2 cells with H-151 treatment. (**K**) IF co-staining of 10 kD, 20 kD or 70 kD FITC-Dextran (green) and DAPI (cyan) following ct or OE-hYAP plasmid transfection. (**L**) IF co-staining of cGAS (red) and DNA (green) in LX-2 cells following ct or OE-hYAP plasmid transfection. Statistical significance: For **F-G**, * P < 0.05, compared with sham group; *^#^ P* < 0.05, *^##^ P* < 0.01, *^###^ P* < 0.001, compared with the TGF-β group.

We then applied electron microscopy to examine the *in vivo* and *in vitro* nuclear envelope ultrastructure in HSCs. The *in vivo* results showed that compared with the regular nuclei in the BDL group, LIG administration resulted in irregular and shriveled nucleus and the disruption of nuclear envelope integrity (**Figure 7A**). Consistent with the *in vivo* findings, *in vitro* electron microscopy analysis of HSC similarly revealed irregular nuclear morphology and disrupted nuclear envelope integrity (**Figure 7B**). To further examine the function of the nucleus, the FITC-Dextran with different molecular weights were used to determine the integrity of nuclear envelope. Incubation of LX-2 cells with 70 kD FITC-labeled dextran resulted in increased nuclear uptake of 70 kD dextran with LIG treatment (**Figure 7C** and **Figure S15A**), confirming that LIG compromised the integrity of the nuclear envelope. Based on the results of DNA IF staining, we confirmed DNA spilling into the cytosol following LIG stimulation (**Figure S15B**). Also, significant nuclear accumulation of γH2AX was observed in LIG20- and LIG40-treated HSCs with TGF-β stimulation, indicating that genomic DNA was cut and released into cytoplasm (**Figure 7D** and **Figure S15C**). However, 40 μM LIG slightly induced nuclear enrichment of γ-H2AX without TGF-β stimulation in LX-2 cells (**Figure S16A**). Several reports suggest that the DNA leakage can bind to cGAS and activate the STING pathway, which further promoted the release of SASPs to induce cellular senescence 28-30. LIG significantly increased the mRNA and protein level of cGAS and STING *in vivo* (**Figure S17A-B**). Additionally, co-staining results of α-SMA and cGAS supported that LIG increased the expression of cGAS in α-SMA-positive HSCs (**Figure S17C-D**). Data from the Human Protein Atlas revealed that STING is mainly expressed in HSCs but barely expressed in hepatocytes (**Figure S17E**). Also, the expression of STING is much higher in HSCs than hepatocytes (**Figure 7E**). These results indicated that STING might play a more important role in HSCs. Furthermore, LIG also significantly increased the mRNA and protein level of cGAS and STING in LX-2 cells (**Figure 7F-G**). Notably, LIG treatment enhanced DNA-cGAS colocalization within the perinuclear cytoplasm of both primary HSCs and LX-2 cells (**Figure 7H-I** and **Figure S18A-B**). However, without TGF-β activation, LIG could not induce DNA leakage and the co-location of DNA and cGAS (**Figure S16B**).

Next, the STING inhibitor, H-151, was used to pretreat LX-2 cells before LIG administration and prevented the HSC senescence induced by LIG (**Figure 7J** and **Figure S18C**). To investigate whether YAP overexpression could counteract LIG's effects, we transfected LX-2 cells with YAP plasmids and applied FITC-labeled dextran again. As expected, the overexpression of YAP failed to conduct 70 kD FITC-labeled dextran entering the nucleus with the treatment of LIG (**Figure 7K** and **Figure S19A**). Likewise, LIG-induced upregulation of STING and cGAS as well as the co-location of DNA and cGAS were reversed with YAP overexpression (**Figure 7L** and **Figure S19B-C**). These results supported that the YAP-regulated dsDNA-cGAS-STING pathway was involved in HSC senescence.

### Either liver-specific knockdown or whole-body knockout of STING abolish the anti-fibrotic effects of LIG in mice

**Figure 8:**
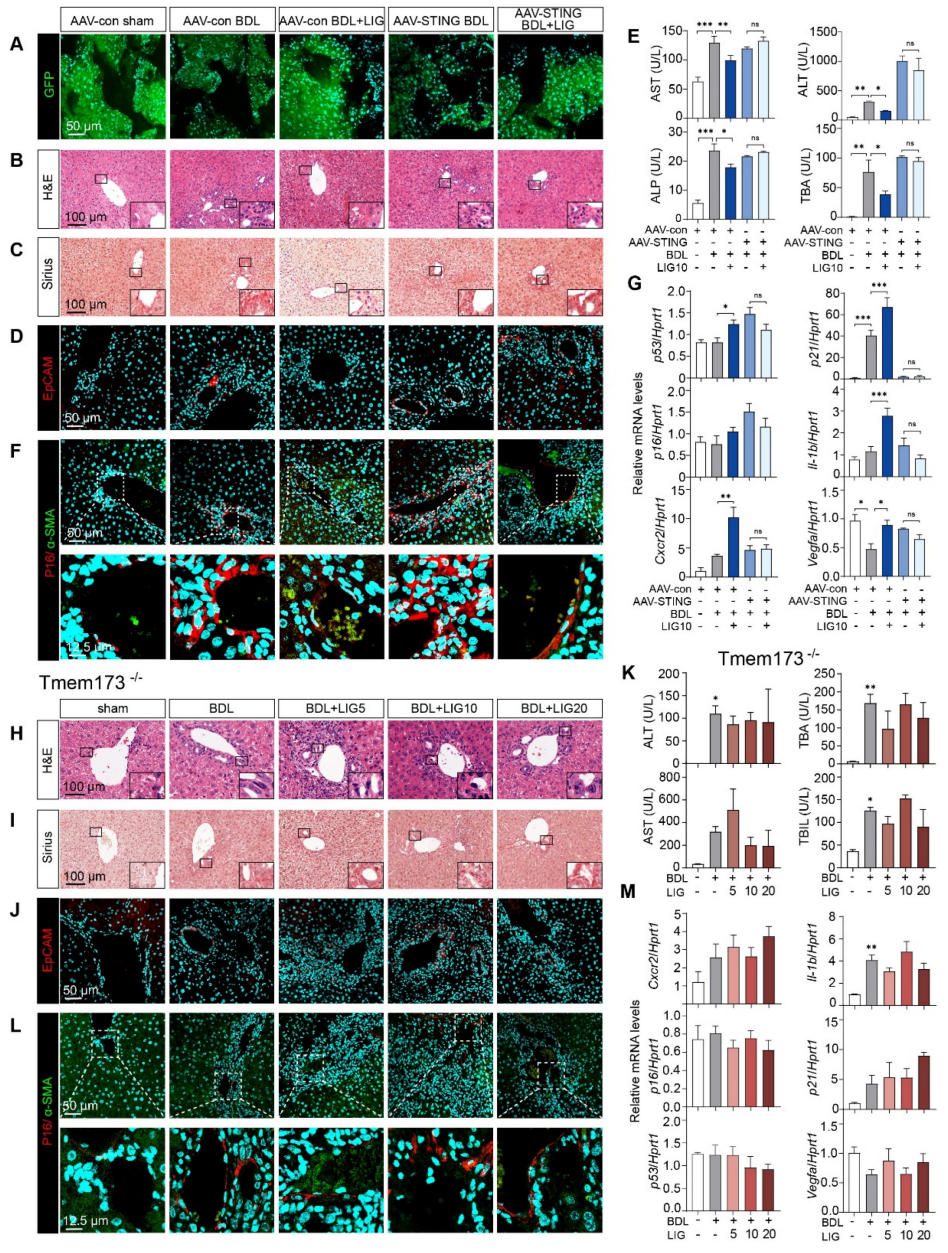
** The knockdown of STING in mouse and the knockout of STING can abolish the anti-fibrotic effects of ligustilide* in vivo*.** (**A**) Staining of GFP from mice with AAV injection. (**B**-**D**) Representative images of H&E (**B**), Sirius red (**C**), IF staining of EpCAM (**D**) from mice with AAV injection. (**E**) Serum levels of biomakers from mice with AAV injection. (**F**) IF co-staining of α-SMA (red) and P16 (green) from mice with AAV injection. (**G**) The mRNA expression of senescence-related genes are determined by qPCR and normalized with *Hprt1* in liver tissues from mice with AAV injection. (**H**-**J**) Representative images of H&E (**H**), Sirius red (**I**), staining of EpCAM (**J**) from Tmem173^-/-^ mice. (**K**) Serum levels of biomakers from Tmem173^-/-^ mice. (**L**) Representative images of IF co-staining of α-SMA (red) and P16 (green) from Tmem173^-/-^ mice. (**M**) The mRNA expression of senescence-related genes are determined by qPCR and normalized with *Hprt1* in Tmem173^-/-^ mice. Statistical significance: * P < 0.05, ** P < 0.01, *** P < 0.001.

Due to the cascade amplification effects inherent in signal transduction, we determined to reverse the STING upregulation* in vivo* and checked whether LIG could still protect against downstream senescence and fibrosis. Thus, we first built liver-specific STING knockdown mice that were achieved using AAV8-shSTING adeno-associated virus and used Tmem173^-/-^ mice for whole-body STING deletion to determine the targeting efficacy of the drug on the liver. Adeno-associated viruses were successfully injected into mice and knockdown the expression of STING in HSCs as evidenced by (**Figure 8A** and **Figure S20A-B)**. Moreover, H&E, Sirius Red and IF staining demonstrated the ameliorations of LIG on liver structural abnormalities, collagen deposition and ductular reactions induced by BDL were abolished in the liver-specific STING knockdown mice (**Figure 8B-D** and **Figure S20C-D**) and Tmem173^-/-^mice (**Figure 8H-J** and **Figure S21A-B**), respectively. Similarly, STING knockdown (**Figure 8E** and **Figure S20E**) or knockout (**Figure 8K** and **Figure S21C**) abolished the suppressive effects of LIG treatment on serum liver function biomarkers and mRNA expression of pro-fibrotic genes. Notably, the co-staining of α-SMA and P16 indicated both the knockdown (**Figure 8F** and **Figure S20F**) or knockout (**Figure 8L** and **Figure S21D**) of STING *in vivo* abolished LIG-induced HSC senescence. Consistently, the senescence-promoting genes and SASPs were unable to be upregulated with LIG administration in these two mouse models (**Figure 8G, M**). Thus, the regulation of LIG on the microfilament rearrangement served as an upstream signal, with its ultimate impact on fibrosis mediated through the YAP-STING pathway in the liver.

## Discussion

Natural products are a significant source of drug candidates, particularly for diseases with limited treatment options. LIG has previously attracted attention for its therapeutic effects on nervous system diseases, cancers and alcoholic hepatic steatosis 21, 31, 32. While our work is the first to report the antifibrotic efficacy of LIG, other studies have confirmed its multifaceted benefits in chronic liver diseases that have not yet developed into fibrosis and malignantly progressive HCC. LIG not only suppresses HCC malignancy but also blocks tumor-associated macrophage recruitment and M2 polarization by inhibiting YAP/IL-6-driven activation of IL-6R/STAT3 signaling 5, suggesting therapeutic utility even in advanced, transformation-prone liver diseases. In addition to fibrosis, LIG ameliorates early-stage CLD. For example. in murine models of alcoholic steatosis, LIG significantly reduces ethanol-induced hepatic lipid accumulation and tissue injury 6. Similarly, LIG alleviated lipid deposition in HepG2 cells and diabetic models with concomitant hepatic protection 7, 8, underscoring its potential as a metabolic dysfunction-associated steatotic liver disease (MASLD) therapeutic. While clinical trials specifically evaluating LIG are lacking, Chuanxiong oil (whose primary ingredients are LIG) has demonstrated efficacy in treating migraine (blood stasis) in the clinic, indicating the clinical application of LIG in the future.

A comprehensive review has summarized the pharmacokinetic and pharmacological properties of LIG, concluding that doses ranging from 5 to 80 mg/kg are safe in mice without causing toxicity 33. Here, we first demonstrated that LIG (10 mg/kg) selectively induces senescence in primary HSCs without affecting hepatocytes. This contradiction is not solely related to the model or disease context. Senescent cells exhibit slow proliferation and metabolism along with cytoskeletal degradation, which is similar to the phenotype of slow-proliferating hepatocytes in the liver 34, 35. Activated HSCs not only proliferate rapidly but also highly express the microfilament network component α-SMA to maintain a dynamic and active microfilament network. Our results indicated that LIG directly binds to G-actin and exerts pro-aging effects by modulating the microfilament network (**Figure 2E-F**). We propose that, owing to differences in actin expression, isoforms and polymerization states across various cell types, the efficacy of LIG in different cell types may vary.

Another study indicated that a dose of 5 mg/kg of LIG effectively prevented thymic epithelial cells (TECs) from undergoing senescence by inhibiting the formation of the actin-Tβ15 complex, which subsequently enhances microfilament assembly in TECs 36. Although this research appears to contradict our findings, it is important to note that, according to The Human Protein Atlas, Tβ15, a member of the β-thymosin family expressed in the thymus, is nearly absent in the liver. Interestingly, our results showed that 5 mg/kg LIG did not promote F-actin depolymerization or HSC senescence. We hypothesize that 5 mg/kg LIG might not be sufficient to bind free G-actin and inhibit F-actin formation. Thus, considering the dynamic remodeling of the microfilament network, LIG exhibits distinct regulatory effects on actively proliferating cells with high versus low F-actin levels. In other word, LIG may have the capacity to induce senescence in excessively activated cells while mitigating senescence in senescent cells, thus acting as a "flexible" molecule with considerable research potential.

Mechanistically, as Rho-MRTF-A-SRF pathway can drive α-SMA expression 9, we firstly suspected that LIG stabilized the actin-MRTF-A interaction by acting as an actin-binding agent, thereby inhibiting nuclear translocation of MRTF-A and reducing its colocalization with SRF in the nucleus. By assessing the changes of key proteins in the expression of key proteins in the Rho-MRTF-A-SRF pathway, we found that LIG significantly inhibited Rho activation but had little effect on SRF expression (**Figure S22A**). Further analysis revealed that LIG slightly enhanced the binding affinity between MRTF-A and actin or reduced nuclear MRTF-A (**Figure S22B-C**). LIG slightly suppressed the expression of SRF downstream targets, including the collagen-producing genes COL1 and α-SMA, in HSCs (**Figure S22D**). However, these effects are relatively weak because the inhibition of COL1 and α-SMA by LIG through the SRF pathway is much weaker than the overall reduction in COL1 and α-SMA levels induced by LIG. Therefore, we further explored the mechanism that responds to LIG-induced microfilament rearrangement.

YAP/TAZ serves as a central transcription factor that connects mechanotransduction and aging, responding to signals from ECM rigidity and cell shape through cytoskeletal mediation 15. Additionally, the interaction between LMNA and F-actin affects nuclear membrane stability. The application of LIG may weaken this interaction and lead to disruption of nuclear membrane integrity (**Figure 6J** and** Figure 7C**). On the other hand, the binding between LMNA and F-actin influences YAP nuclear-cytoplasmic translocation of YAP. Both the depolymerization of F-actin and the reduction in LMNA decrease the nuclear distribution of YAP 37, 38, which may explain why LIG induces the translocation of YAP translocation from the nucleus to the cytoplasm. Notably, we also found that the overexpression of YAP reversed the LIG-induced downregulation of LMNA expression (**Figure 6G, I**), suggesting that LMNA might be a downstream gene regulated by YAP. Additionally, the Cistrome DB database was used to predict the downstream targets of YAP, which identified LMNA as a downstream target of YAP. Consequently, decreased nuclear membrane permeability led to reduced LMNA expression caused by YAP nuclear leakage, which in turn disrupted nuclear membrane structure and created a positive feedback loop that enhanced the effects of LIG on nuclear membrane integrity. Moreover, studies have shown that directly silencing YAP in activated HSCs induces the expression of the senescence-associated marker P21 and promotes senescence in activated HSCs, thereby improving liver fibrosis 39. These studies support the conclusion that HSC senescence induced by LIG was attributed to reduced YAP nuclear localization.

In recent years, the crucial role of the cGAS-STING pathway in digestive system diseases has been increasingly recognized 40, 41. During the aging process, the production of cGAMP increases, leading to aberrant activation of cGAS-STING and subsequent phosphorylation of the downstream TBK1 42. STING-IRF3-RB signaling plays a notable role in HSCs by promoting the expression of P16 and secretory factors associated with the SASP, thereby driving activated HSCs into senescence. Recent research has identified that in stromal cells, decreased YAP/TAZ mechanotransduction drives aging by activating cGAS-STING signaling. The decrease in nuclear YAP/TAZ led to reduced expression of its target genes actin-related protein 2 (ACTR2) and LMNB1, compromising nuclear membrane integrity and exposing DNA to the cytoplasm, thereby activating the downstream cGAS-STING signaling pathway and the SASP 8. As YAP has been shown to control the cGAS-STING pathway, our study revealed a novel mechanism by which microfilaments mediate the YAP-dependent cGAS-STING pathway to induce HSC senescence. Owing to the cascading effects of this signaling pathway, we hypothesized that the upregulation of STING acts as a key inducer of HSC senescence. Therefore, we utilized two *in vivo* animal models to demonstrate that liver-specific knockdown or systemic knockout of STING can reverse LIG-induced HSC senescence and ameliorate liver fibrosis. Interestingly, we found that compared with systemic knockdown of STING, liver-specific knockdown of STING strongly reversed the efficacy of LIG. Although there was no significant difference between the Tmem173^-/-^ mice treated with BDL + LIG and those treated with BDL alone, we observed a trend toward reduced elevation of the serum TBA and AST levels following LIG treatment. Moreover, the specific knockdown of STING in the liver nearly completely abolished the upregulation of *p16*, whereas the whole-body knockout of STING did not have as much of an effect in the liver. We hypothesize that, on the basis of the crucial role of STING signaling in both innate and adaptive immunity 40, whole-body knockout of STING might interrupt the immune process to some extent, increasing complexity to this phenomenon. Our results indicate that LIG may not directly regulate the STING pathway; instead, it influences this pathway indirectly through the modulation of microfilament rearrangement, indicating that LIG may offer advantages over traditional STING agonists.

We summarized the reasons why LIG has distinct effects on different cells and how these effects related to senescence in two points: (1) Regarding upstream mechanisms, the F-actin staining results of primary HSCs and hepatocytes revealed that cytoskeletal remodeling in activated HSCs was significantly more active than that in hepatocytes (**Figure 2B-C**). Additionally, we demonstrated that high α-SMA expression is correlated with HSC activity. Thus, the microfilament depolymerization induced by LIG triggered a stronger downstream cascade response in HSCs. (2) Regarding downstream signaling, we found that STING was highly expressed in HSCs, whereas hepatocytes presented minimal STING expression (**Figure 7E**). These findings suggest that nuclear dsDNA leakage in HSCs activated the STING pathway, promoting senescence more prominently. Moreover, our findings indicated that LIG alone, whether administered *in vivo* or *in vitro*, did not induce senescence (**Figure S7** and **S16**), which indicates that the microfilament depolymerization mediated by LIG served as an upstream mechanism for senescence induction.

In summary, LIG disrupted the microfilament rearrangement by directly interacting with G-actin, which further compromised the integrity of the nuclear membrane maintained by F-actin and LMNA. As a result, YAP is translocated from the nucleus to the cytoplasm, leading to a reduction in LMNA expression. The release of leaked dsDNA subsequently activated the cGAS-STING pathway, leading to the release of secretion factors associated with the SASP, HSC senescence, and ultimately attenuated liver fibrosis. These findings underscore the potential of specifically targeting the hepatic microfilaments as a promising therapeutic strategy.

## Supplementary Material

Supplementary methods, figures and tables.

## Abbreviations

ALT: alanine aminotransferase
AST: aspartate aminotransferase
cGAS: cyclic-GMP-AMP synthase
Cxcr2: C-X-C motif chemokine receptor 2
dsDNA: double-stranded DNA
ECM: extracellular matrix
F-actin: polymeric filamentous actin
G-actin: globular actin
GFAP: glial fibrillary acidic protein
H&E: hematoxylin and eosin
HSC: hepatic stellate cell
Il-1b: interleukin 1 beta
LIG: ligustilide
LMNA: lamin A/C
p16: cyclin dependent kinase inhibitor 2A
p21: cyclin dependent kinase inhibitor 1A
SASP: senescence-associated secretory phenotype
STING: stimulator of interferon gene
TBA: total bile acid
TBIL: total bilirubin
TGF-β: transforming growth factor
p53: tumor protein p53
UDCA: ursodeoxycholic acid
Vegfa: vascular endothelial growth factor A
YAP: yes-associated protein

## References

[1] Hammerich L, Tacke F (2023). Hepatic inflammatory responses in liver fibrosis. Nat Rev Gastroenterol Hepatol.

[2] Tsuchida T, Friedman SL (2017). Mechanisms of hepatic stellate cell activation. Nat Rev Gastroenterol Hepatol.

[3] Krizhanovsky V, Yon M, Dickins RA, Hearn S, Simon J, Miething C (2008). Senescence of activated stellate cells limits liver fibrosis. Cell.

[4] Wang F, Li Z, Chen L (2022). Inhibition of ASCT2 induces hepatic stellate cell senescence with modified proinflammatory secretome through an IL-1 α/NF- κ B feedback pathway to inhibit liver fibrosis. Acta Pharm Sin B.

[5] Yashaswini CN, Qin T, Bhattacharya D, Amor C, Lowe S, Lujambio A (2024). Phenotypes and ontogeny of senescent hepatic stellate cells in metabolic dysfunction-associated steatohepatitis. J Hepatol.

[6] Zhang M, Serna-Salas S, Damba T, Borghesan M, Demaria M, Moshage H (2021). Hepatic stellate cell senescence in liver fibrosis: Characteristics, mechanisms and perspectives. Mech Ageing Dev.

[7] Sun Y, Weng J, Chen X, Ma S, Zhang Y, Zhang F (2023). Oroxylin A activates ferritinophagy to induce hepatic stellate cell senescence against hepatic fibrosis by regulating cGAS-STING pathway. Biomed Pharmacother.

[8] Sladitschek-Martens HL, Guarnieri A, Brumana G, Zanconato F, Battilana G, Xiccato RL (2022). YAP/TAZ activity in stromal cells prevents ageing by controlling cGAS-STING. Nature.

[9] Chang Z, Li L-Y, Shi Z-J, Liu W, Xu G-K (2024). Beyond stiffness: Multiscale viscoelastic features as biomechanical markers for assessing cell types and states. Biophysical Journal.

[10] Chang Z, Zhang L, Hang JT, Liu W, Xu GK (2023). Viscoelastic Multiscale Mechanical Indexes for Assessing Liver Fibrosis and Treatment Outcomes. Nano Lett.

[11] Qu J, Qiu B, Zhang Y, Hu Y, Wang Z, Guan Z (2023). The tumor-enriched small molecule gambogic amide suppresses glioma by targeting WDR1-dependent cytoskeleton remodeling. Signal Transduct Target Ther.

[12] Kai F, Leidal AM, Weaver VM (2025). Tension-induced organelle stress: an emerging target in fibrosis. Trends Pharmacol Sci.

[13] Davidson PM, Cadot B (2021). Actin on and around the Nucleus. Trends Cell Biol.

[14] Gunasekaran S, Miyagawa Y, Miyamoto K (2022). Actin nucleoskeleton in embryonic development and cellular differentiation. Curr Opin Cell Biol.

[15] Dupont S, Morsut L, Aragona M, Enzo E, Giulitti S, Cordenonsi M (2011). Role of YAP/TAZ in mechanotransduction. Nature.

[16] Qu J, Xue X, Wang Z, Ma Z, Jia K, Li F (2024). Si-Wu-Tang attenuates liver fibrosis via regulating lncRNA H19-dependent pathways involving cytoskeleton remodeling and ECM deposition. Chin J Nat Med.

[17] Qu J, Wang L, Li Y, Li X (2024). Liver sinusoidal endothelial cell: An important yet often overlooked player in the liver fibrosis. Clin Mol Hepatol.

[18] Takaya K, Kishi K (2024). Ligustilide, A Novel Senolytic Compound Isolated from the Roots of Angelica Acutiloba. Adv Biol (Weinh).

[19] Li JJ, Zhu Q, Lu YP, Zhao P, Feng ZB, Qian ZM (2015). Ligustilide prevents cognitive impairment and attenuates neurotoxicity in D-galactose induced aging mice brain. Brain Res.

[20] Zhu WL, Zheng JY, Cai WW, Dai Z, Li BY, Xu TT (2020). Ligustilide improves aging-induced memory deficit by regulating mitochondrial related inflammation in SAMP8 mice. Aging (Albany NY).

[21] Yang HX, Jiang XL, Zuo RM, Wu YL, Nan JX, Lian LH (2024). Targeting RXFP1 by Ligustilide: A novel therapeutic approach for alcoholic hepatic steatosis. Int Immunopharmacol.

[22] Hirschfield GM, Dyson JK, Alexander GJM, Chapman MH, Collier J, Hübscher S (2018). The British Society of Gastroenterology/UK-PBC primary biliary cholangitis treatment and management guidelines. Gut.

[23] Chen W, Yang A, Jia J, Popov YV, Schuppan D, You H (2020). Lysyl Oxidase (LOX) Family Members: Rationale and Their Potential as Therapeutic Targets for Liver Fibrosis. Hepatology.

[24] Chen X, Li WX, Chen Y, Li XF, Li HD, Huang HM (2018). Suppression of SUN2 by DNA methylation is associated with HSCs activation and hepatic fibrosis. Cell Death Dis.

[25] Rockey DC, Du Q, Weymouth ND, Shi Z (2019). Smooth Muscle α-Actin Deficiency Leads to Decreased Liver Fibrosis via Impaired Cytoskeletal Signaling in Hepatic Stellate Cells. Am J Pathol.

[26] Tiwari V, Alam MJ, Bhatia M, Navya M, Banerjee SK (2024). The structure and function of lamin A/C: Special focus on cardiomyopathy and therapeutic interventions. Life Sci.

[27] Kim JK, Louhghalam A, Lee G, Schafer BW, Wirtz D, Kim DH (2017). Nuclear lamin A/C harnesses the perinuclear apical actin cables to protect nuclear morphology. Nat Commun.

[28] Wu Q, Leng X, Zhang Q, Zhu YZ, Zhou R, Liu Y (2024). IRF3 activates RB to authorize cGAS-STING-induced senescence and mitigate liver fibrosis. Sci Adv.

[29] Yu L, Liu P (2024). cGAS/STING signalling pathway in senescence and oncogenesis. Semin Cancer Biol.

[30] Ruiz de Galarreta M, Lujambio A (2017). DNA sensing in senescence. Nat Cell Biol.

[31] Li XX, Yang P, Zhang YH, Song W, Wang XM, Li J (2024). Research progress on mechanisms of ligustilide in treatment of nervous system diseases. Zhongguo Zhong Yao Za Zhi.

[32] Wu Y, Rong L, Zhang S, He Y, Song N, Zuo G (2025). Ligustilide Inhibits the PI3K/AKT Signalling Pathway and Suppresses Cholangiocarcinoma Cell Proliferation, Migration, and Invasion. Recent Pat Anticancer Drug Discov.

[33] Xie Q, Zhang L, Xie L, Zheng Y, Liu K, Tang H (2020). Z-ligustilide: A review of its pharmacokinetics and pharmacology. Phytother Res.

[34] Michalopoulos GK (2017). Hepatostat: Liver regeneration and normal liver tissue maintenance. Hepatology.

[35] Lai W-F, Wong W-T (2020). Roles of the actin cytoskeleton in aging and age-associated diseases. Ageing Res Rev.

[36] Xu X, Tao N, Sun C, Hoffman RD, Shi D, Ying Y (2024). Ligustilide prevents thymic immune senescence by regulating Thymosin β15-dependent spatial distribution of thymic epithelial cells. Phytomedicine.

[37] Shiu JY, Aires L, Lin Z, Vogel V (2018). Nanopillar force measurements reveal actin-cap-mediated YAP mechanotransduction. Nat Cell Biol.

[38] Wu T, Zheng F, Tang HY, Li HZ, Cui XY, Ding S (2024). Low-intensity pulsed ultrasound reduces alveolar bone resorption during orthodontic treatment via Lamin A/C-Yes-associated protein axis in stem cells. World J Stem Cells.

[39] Du K, Maeso-Díaz R, Oh SH, Wang E, Chen T, Pan C (2023). Targeting YAP-mediated HSC death susceptibility and senescence for treatment of liver fibrosis. Hepatology.

[40] Li XJ, Qu JR, Zhang YH, Liu RP (2024). The dual function of cGAS-STING signaling axis in liver diseases. Acta Pharmacol Sin.

[41] Cai Y, Li S, Yang Y, Duan S, Fan G, Bai J (2024). Intestinal epithelial damage-derived mtDNA activates STING-IL12 axis in dendritic cells to promote colitis. Theranostics.

[42] Gulen MF, Samson N, Keller A, Schwabenland M, Liu C, Glück S (2023). cGAS-STING drives ageing-related inflammation and neurodegeneration. Nature.

