# Supplementary material for: Disturbance of cytoskeleton induced by ligustilide promotes hepatic stellate cell senescence and ameliorates liver fibrosis
Source: Theranostics. 2025 Jul 24;15(16):8049–67. doi: 10.7150/thno.108869 (PMC12374544; doi:10.7150/thno.108869)
Supplement: Supplementary file 1 — Supplementary methods, figures and tables. [file thnov15p8049s1.pdf]

Supplemental Digital Content

1. Supplementary methods

1.1 Cell culture

The human HSCs, LX-2 cells were obtained from the Cell Bank of Chinese Academy of Sciences (Shanghai Institutes for Biological Sciences, Chinese Academy of Sciences). The rat HSCs, HSC-T6 cells were obtained from BNCC (BNCC341155, BNCC, Beijing, China). Primary mouse hepatocyte and HSCs were isolated according to a method we established before. LX-2 cells were cultured with 1640 medium supplemented with 10% fetal bovine serum (FBS) and 1% penicillin-streptomycin solution, which was activated with 5 μg/mL TGF-β. HSC-T6 cells, primary HSCs and primary hepatocyte were cultured in DMEM supplemented with 10% FBS and 1% penicillin-streptomycin solution in a incubator containing 5% CO_2 at 37 ℃. HSC-T6 cells were activated with 10 μg/mL PDGF-BB. For transfection experiments, control plasmid and YAP plasmid were transfected into LX-2 cells using Lipofectamine 3000.

1.2 Quantitative real-time RT-PCR (qPCR)

The total RNA from liver tissues and cells were extracted by trizol reagent. According to the measured concentration, the total RNA was reversed to obtain more stable cDNA. Then the mRNA expression was detected by quantitative real-time PCR (qPCR) and calculated according to the expression of the reference gene. The detailed primer sequences were shown in **Supplementary table 1**.

1.3 Western blot (WB) analysis

Proteins from cells and liver tissues were lysed using RIPA lysis buffer. Then the proteins were separated on a 10% SDS-PAGE gel, transferred to the PVDF membranes and blocked with 5% BSA TBST buffer for 1 h. The membranes were incubated overnight with the specific primary antibodies listed in Table S1. After incubation with secondary antibodies, the protein bands were visualized using ECL reagents.

1.4 Immunofluorescence (IF) staining

For cells, after washing with PBS, cells were fixed with 4% paraformaldehyde

(PFA) for 30 min and permeabilized with 1%BSA PBS solution containing 0.1%Triton X-100 for 30 min. For tissues, the tissues paraffin sections were deparaffinized first and boiled in 85 ℃ boiling antigen recovery solution for 40 min. After cooling at room temperature, the tissues were blocked by adding blocking solution containing 10% goat serum for 30 min.

Then the primary antibody was incubated in a 4 ℃ refrigerator overnight. Finally, followed by incubation of corresponding secondary antibodies at room temperature and staining with DAPI, cells or tissues were observed with Olympus FV3000 confocal laser scanning microscopy (Tokyo, Japan). Primary and secondary antibodies were listed in Table S1.

1.5 Immunohistochemical staining and histopathologic analysis

For immunohistochemical staining, the tissues paraffin sections were deparaffinized first and boiled in 85 ℃ boiling antigen recovery solution for 40 min. After cooling at room temperature, using the hydrogen peroxide in IHC detect kit (PK10006, Proteintech, USA) incubation for 30 min, then the tissues were blocked by adding blocking solution containing 10% goat serum for 30 min. Then the primary antibody was incubated in a 4 ℃ refrigerator overnight. After incubation with a universal secondary antibody, DAB staining, hematoxylin counterstained, and dehydration, the tissues were mounted with neutral gum and allowed to dry before being imaged.

Liver tissues from mice were immersed in formalin for 7 days, dehydrated in ethanol, embedded in paraffin and made into sections. Sections were deparaffinized with xylene, various concentrations of ethanol, and water and stained with hematoxylin and eosin (H & E). All images were taken by Aperio Versa (Laika, Wetzlar, Germany).

1.6 Senescence-associated-β-galactosidase (SA-β-Gal) staining

The LX-2 cells were plated in a 6-well plate and stained using the senescence-associated β-galactosidase (SA-β-Gal) stain kit (G1580, Solarbio, Beijing, China) to detect cell senescence, following the kit's instructions. Briefly, the living cells were washed once by adding PBS and were fixed by adding β-Gal fixative solution at room temperature for 15 min, and then washed twice by PBS, and incubated for 48 h in an

incubator without carbon dioxide at 37 ℃ with staining solution. Finally, the senescent cells, which exhibited blue staining, were then photographed under a microscope.

1.7 EdU click assay

Firstly, EdU was added and incubated with cells for 30 min. After washing with PBS, the cells were fixed with paraformaldehyde, and then permeabilized with 0.3% triton X-100 in PBS for 15 min. EdU click-reaction solution was prepared according to the instructions, added to cells and then incubated at room temperature in the dark for 30 min. Finally, DAPI was used to stain the nucleus. The proliferation of the cells could be observed by imaging under a confocal microscope.

1.8 Nuclear and cytoplasmic protein separation assay

The nuclear and cytoplasmic proteins were separated by nuclear and cytoplasmic protein extraction kit (R0050, Solarbio, Beijing, China). Firstly, preparation of cytoplasmic and nuclear protein lysate for use. 80 μl of cytosolic lysate was added to each culture dish on ice for 30 min. Then, cells were scraped with cell scrapers, and after centrifugation the supernatant was cytosolic proteins. 50 μl of nuclear protein lysate was added to the precipitate and vortexed every 10 min for a total of 4 times to fully dissolve. After centrifugation, the supernatant was nucleoprotein. Finally, the expression of protein level was detected by WB.

1.9 Transmission electron microscopy (TEM)

Liver tissue and cell was fixed using an electron microscopy fixative and preserved at 4 ℃. Subsequently, the tissue was washed with phosphate-buffered saline (PBS, 0.1 M, pH 7.4) and fixed in 1% osmium tetroxide (OsO4) in 0.1 M PBS for 2 h at room temperature. The tissue was then dehydrated in ethanol and embedded in resin. The resin-embedded tissue was polymerized in an oven at 65 ℃ for 48 h and subsequently cut into 60 nm thin sections. These sections were examined using transmission electron microscopy (HT7800, Hitachi) to analyze the characteristics of the nucleus in HSCs.

2. Supplementary table 1

Primers for qPCR	Sense (5'-3')	Sense (3'-5')
h-ACTA2 (for chip-qPCR)	GGAACGAGTACCACCAACCC	CTGTAAGTCGCTGCCTGAGT
h-ANXA2	ACATTGAAACAGCCATCAAGAC	GAAGGCAATATCCTGTCTCTGT
h-CCND1	GTCCTACTTCAAATGTGTGCAG	GGGATGGTCTCCTTCATCTTAG
h-CDK2	CCTGGGCTGCAAATATTATTCC	TGGCTTGTAATCAGGCATAGAA
h-CGAS	GCTGCTCCACGAAGCCAAGAC	GCGGCTGAGCTTCAACTTCTCC
h-COL1	GGCTCCTGCTCCTCTTAG	GGATGTCTTCGTCTTGGC
h-COL1 (for chip-qPCR)	CCACAGCCATGGCAAACAAA	GGACACAGAGGTGGGGATTG
h-FN	AATAGATGCAACGATCAGGACA	GCAGGTTTCCTCGATTATCCTT
h-IGF2BP2	CCCTAGTCAACACAGACACAGA	AGCTTCTCCATGGCTATTTTTGC
h-IL-1β	GCCAGTGAAATGATGGCTTATT	AGGAGCACTTCATCTGTTTAGG
h-IL6	AGACAGCCACTCACCTCTTCAG	TTCTGCCAGTGCCTCTTTGCTG
h-LMNA	CAAGACCCTTGACTCAGTAGC	TTCAGCTCCTTAAACTCCTCAC
h-NRG1	CTTTCAAACCCCTCGAGATACT	TGTTTCTTGGTTTTGCAGTAGG
h-P53	TTCCTGAAAACAACGTTCTGTC	AACCATTGTTCAATATCGTCCG

h-STING	GCCCTGTTGCTGCTGTCC ATC	GGATGTTCAGTGCCTGCG AGAG
h-VEGFA	ATCGAGTACATCTTCAAGC CAT	GTGAGGTTTGATCCGCATA ATC
h-YAP	GAACAATGACGACCAATA GCTC	TAGTCCACTGTCTGTACTC TCA
m-Acta2	GTCATCCACAGACAGAGT AGG	CTCCCAACAGACCTGTCTA TAC
m-Cgas	AGGAACCCTGAAGAAATC TCTGTGG	CCAGCCAGCCTTGAATAG GTAGTC
m-Cxcr2	GGGCTGCATCTAAAGTAA ATGG	CAGAACACTGCTGTAGAA GGTA
m-Fn	CTATAGGATTGGAGACAC GTGG	CTGAAGCACTTTGTAGAGC ATG
mGfap	AGATTCGCACTCAATACGA GG	CTGTGAGGTCTGCAAACTT AGA
m-Il-1β	AATCTCACAGCAGCACATC	AGCAGGTTATCATCATCAT CC
m-Lmna	CCTTCGCACCGCTCTCAT CAAC	TCTTCTCCATCCTCGTCGT CATCC
m-Lrat	GAGCAGACATCCTAGTCA ATCA	AGAGCCATATCTGCAGTAA GTC
m-P16	TCAAGACATCGTGCGATAT TTG	TTAGCTCTGCTCTTGGGAT TG
m-P21	ATGTCCAATCCTGGTGATG TC	GAAGTCAAAGTTCCACCGT TC
m-P53	TGGAAGGAAATTTGTATCC CGA	GTGGATGGTGGTATACTC AGAG

m-Spp1	AAACACACAGACTTGAGC ATTC	TTAGGGTCTAGGACTAGCT TGT
m-Sting	GGTCACCGCTCCAAATAT GTAG	CAGTAGTCCAAGTTCGTG CGA
m-Timp1	GCAAAGAGCTTTCTCAAAG ACC	CTCCAGTTTGCAAGGGATA GAT
m-Vegfa	TAGAGTACATCTTCAAGCC GTC	CTTTCTTTGGTCTGCATTC ACA
r-Acta2	AGGGAGTGATGGTTGGAA TGGG	GGTGATGATGCCGTGTTC TATCG
r-Ccnd1	GAGGCGGATGAGAACAAG CAGATC	GGAGGGTGGGTTGGAAAT GAACTTC
r-Col1	ATCCTGCCGATGTCGCTAT CC	TTCTTGAGGTTGCCAGTCT GTTG
r-Fn	AGGCACAAGGTCCGAGAA GAG	TGAGTCATCCGTAGGCTG GTTC
r-IL6	GAGGGCTTCCTAGACACT CTGGTAG	AGATACCGCAAATACCGC ACGAC
r-P21	TCCTGGTGATGTCCGACC TGTTC	GGCTCAACTGCTCACTGT CCAC

Supplementary table 2

Antibodies	Source	Identifier
EpCAM	proteintech	21050-1-AP
COL1	proteintech	67288-1-Ig
FN	proteintech	15613-1-AP
α-SMA	CST	19245S
ALB	proteintech	16475-1-AP
LYVE-1	CST	67538
GFAP	proteintech	16825-1-AP
P16	SantaCruz	sc-56330
P53	proteintech	60283-2-Ig
P21	proteintech	10355-1-AP
β-actin	proteintech	66009-1-Ig
GAPDH	proteintech	60004-1-Ig
LMNA	proteintech	10298-1-AP
YAP	CST	14074T
p-YAP	CST	13008
Lamin B	proteintech	66095-1-Ig
STING	proteintech	19851-1-AP
p-STING	CST	50907
cGAS	proteintech	26416-1-AP
H2AX	proteintech	29380-1-AP
Anti-DNA	Millpore	CBL186

3. Supplement figures

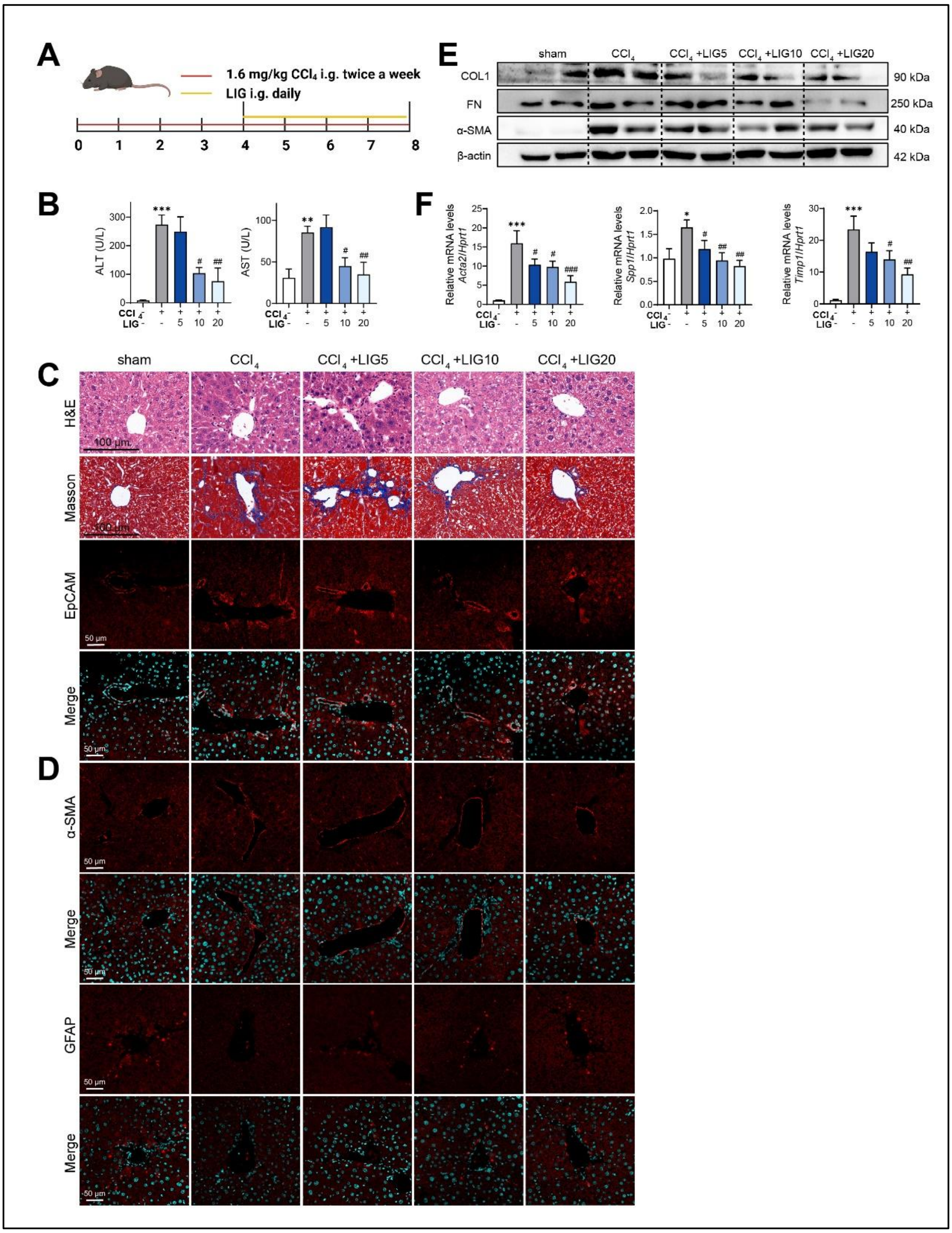

Figure S1. LIG ameliorates CCl_4-induced liver fibrosis. (**A**) The diagram of mice model. (**B**) Serum levels. (**C**) Representative images of H&E, Masson staining and IF staining for EpCAM. (**D**) IF staining for α-SMA and GFAP. (**E**) Protein levels of COL1, FN, and α-SMA were evaluated by WB and normalized to β-actin. (**F**) Relative mRNA

levels of *Acat2*, *Spp1*, and *Timp1* were determined by qPCR and normalized to *Hprt1*. Statistical significance: * $P < 0.05$, ** $P < 0.01$, *** $P < 0.001$, compared with sham group; # $P < 0.05$, ## $P < 0.01$, ### $P < 0.001$, compared with the CCl_4 group.

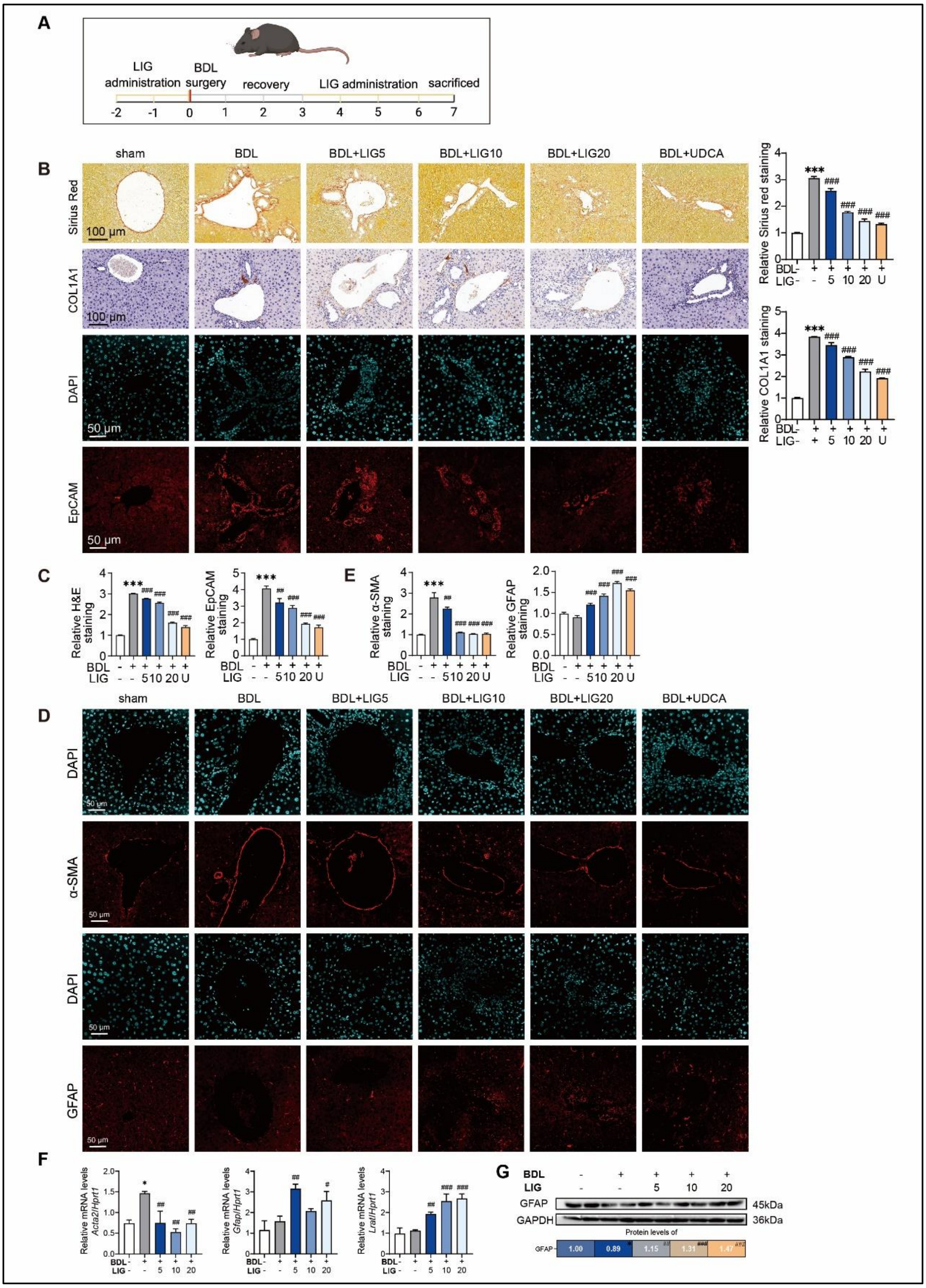

Figure S2. LIG ameliorates BDL-induced liver fibrosis. (**A**) The diagram of mice model. (**B**) Representative images of Sirius red staining, IHC staining of COL1A1 and IF staining for EpCAM. The quantification results are shown in the right. (**C**) The quantification results of H&E and EpCAM staining. (**D**) Representative staining images

of α-SMA and GFAP. (**E**) The quantification results of α-SMA and GFAP staining. (**F**) Relative mRNA levels of *Acta2*, *Gfap*, and *Lrat* were assessed by qPCR and normalized to *Hprt1*. (**G**) Protein levels of GFAP were measured by WB and normalized to GAPDH. Statistical significance: * $P < 0.05$, *** $P < 0.001$, compared with sham group; # $P < 0.05$, ## $P < 0.01$, ### $P < 0.001$, compared with the BDL group.

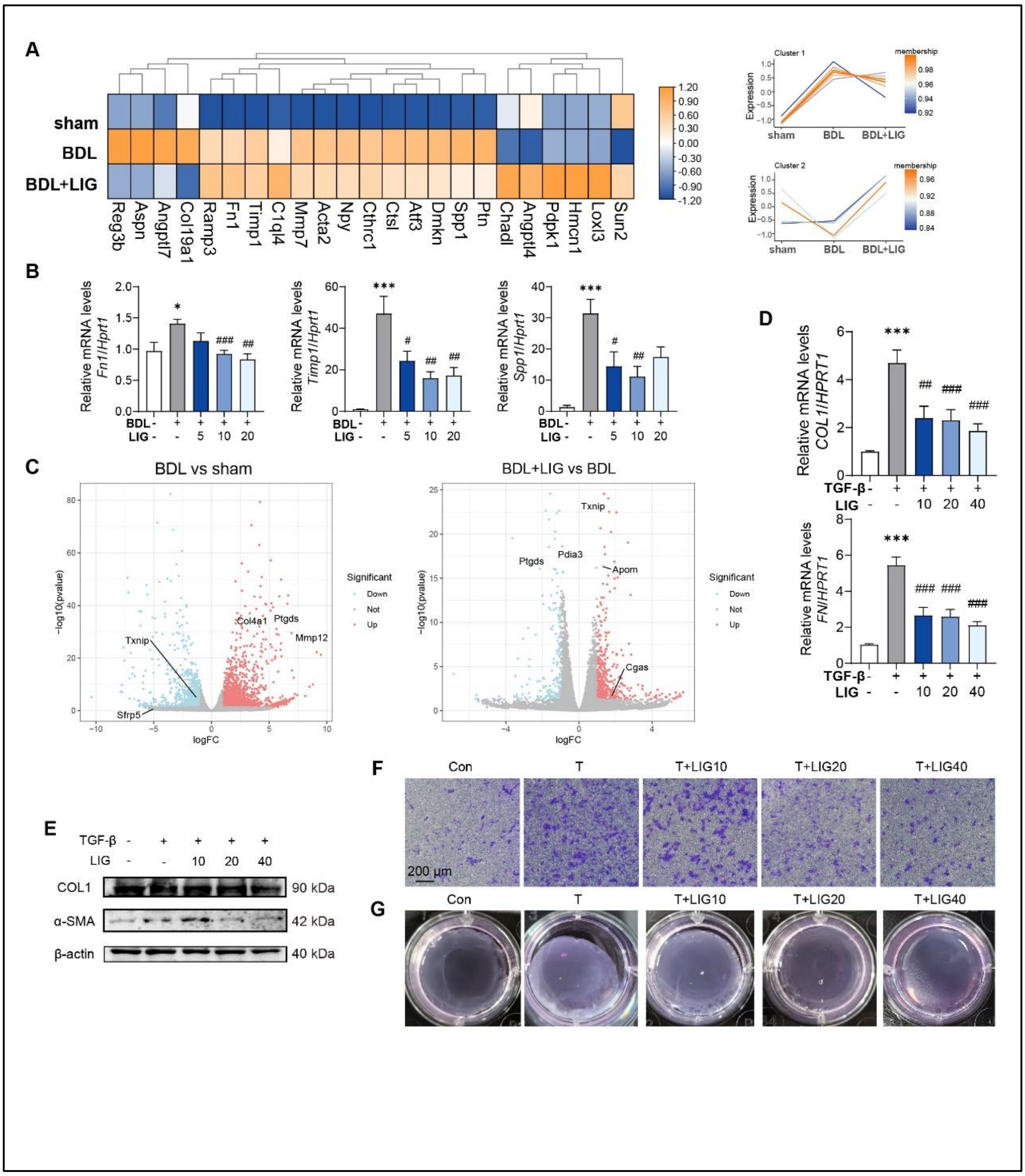

Figure S3. Ligustilide ameliorates experimental liver fibrosis and regulates HSC activity. (**A**) Heatmap and clustering of fibrosis-related marker genes. (**B**) Relative mRNA levels of *Timp1*, *Fn1*, and *Spp1* were assessed by qPCR and normalized to *Hprt1*. (**C**) Volcano plots of RNA-seq analysis for BDL group vs sham group and BDL + LIG group vs BDL group. (**D**) Relative mRNA levels of *COL1* and *FN* were determined in LX-2 cells by qPCR and normalized with *HPRT1*. (**E**) The protein levels of COL1 and α-SMA were determined in LX-2 cells by WB and normalized by β-actin. (**F**) Representative images of transwell migration. (**G**) Representative images of collagen gel. Statistical significance: *P < 0.05, **P < 0.01, *** P < 0.001, compared with the

sham group; # $P < 0.05$, ## $P < 0.01$, ### $P < 0.001$, compared with the BDL group.

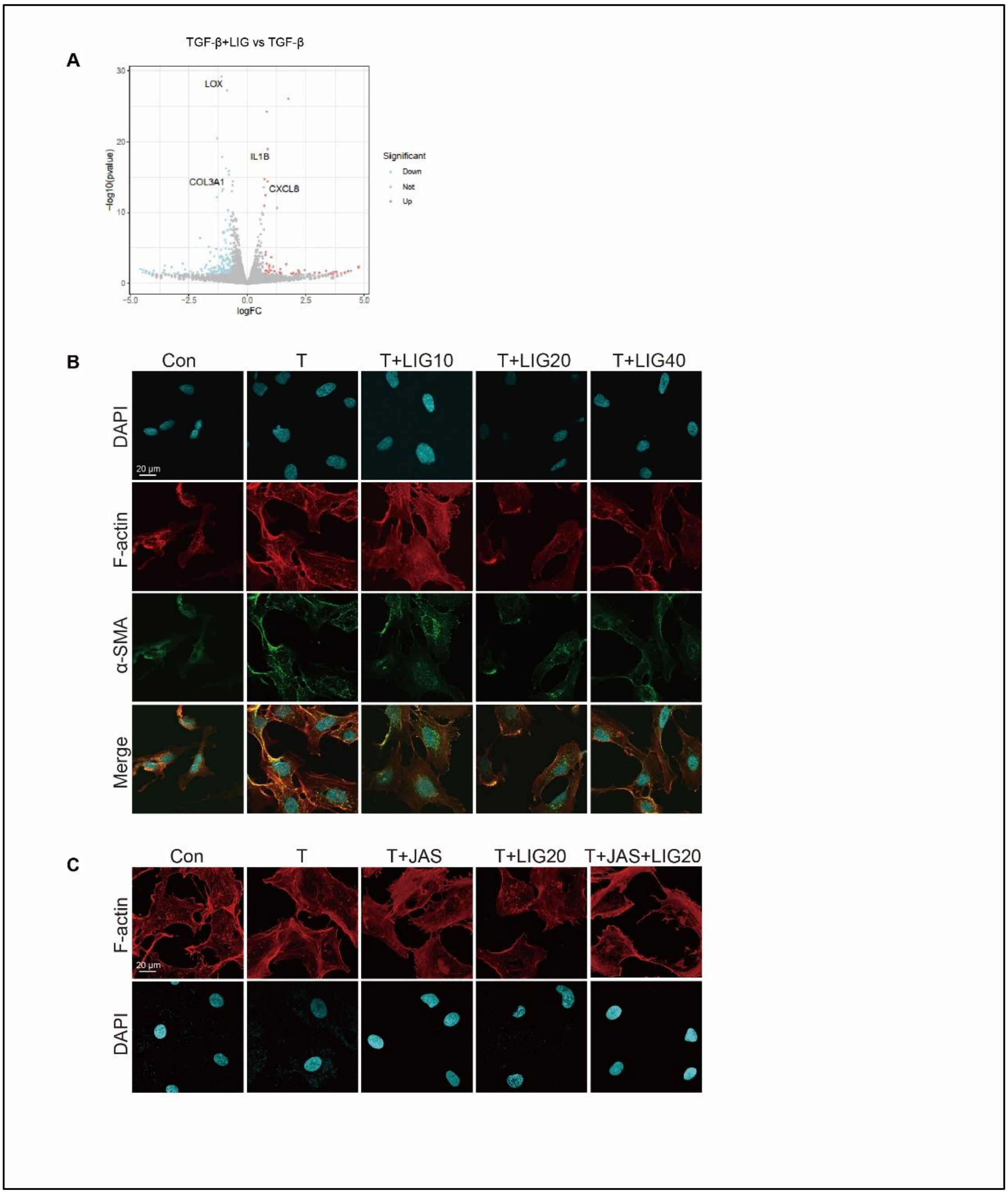

Figure S4. LIG promotes the cytoskeleton depolymerization in HSCs. (**A**) Volcano plots of the DEGs from cellular RNA-seq analysis between the TGF-β group and TGF-β + LIG group. (**B**) Representative images of IF staining for α-SMA, F-actin and DAPI in LX-2 cells. (**C**) Representative images of IF staining for F-actin (red) and DAPI (cyan) images in LX-2 cells after treating with JAS.

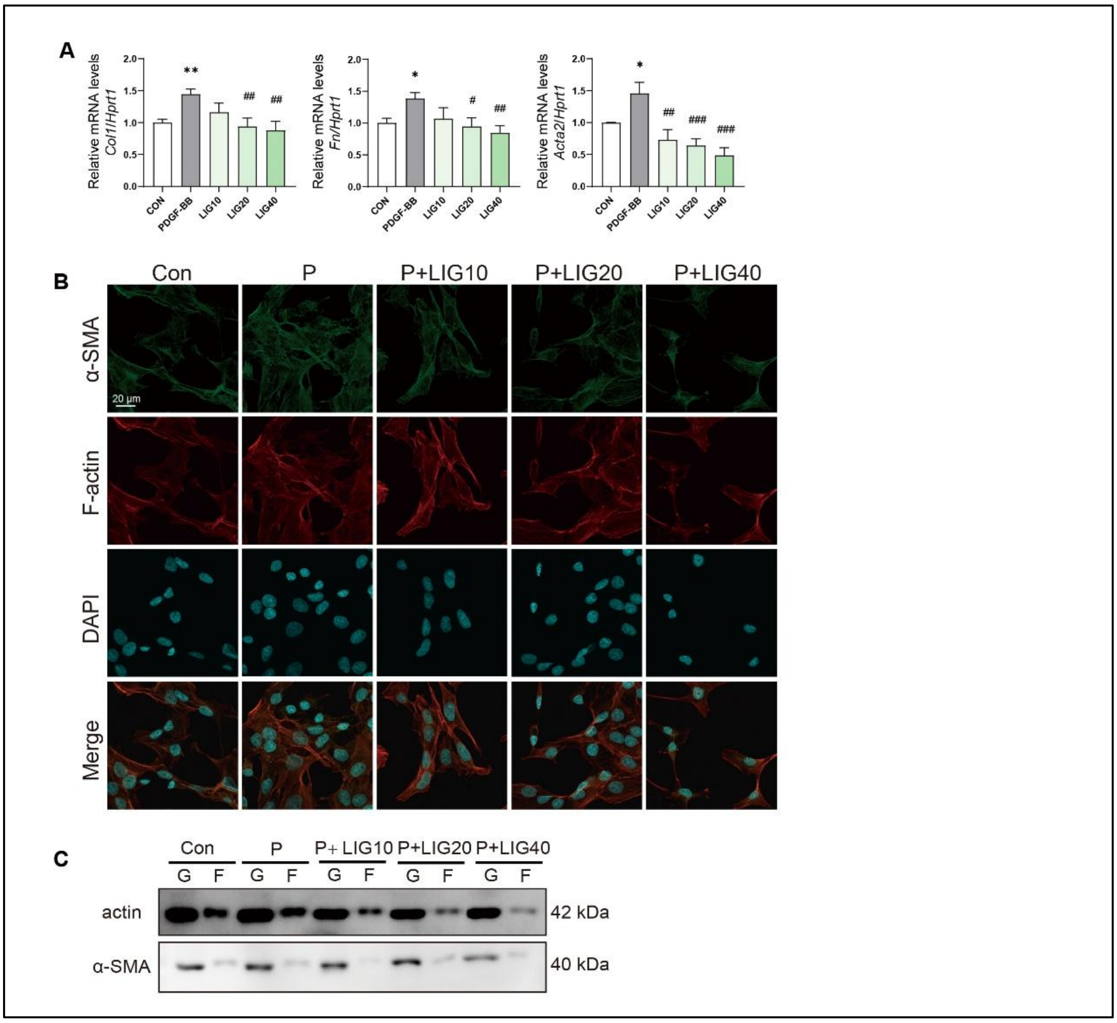

Figure S5. LIG promotes the cytoskeleton depolymerization in HSC-T6 cells. (**A**) Relative mRNA levels of *Col1*, *Fn* and *Acta2* were determined in HSC-T6 cells by qPCR and normalized with *Hprt1*. (**B**) Representative images of IF co-staining of α-SMA, F-actin and DAPI (cyan) in HSC-T6 cells. (**C**) F-actin and G-actin extracted from HSC-T6 cells treated with PDGFBB (20 ng/ml) or LIG for 24 h are subjected to western blot with actin and α-SMA.

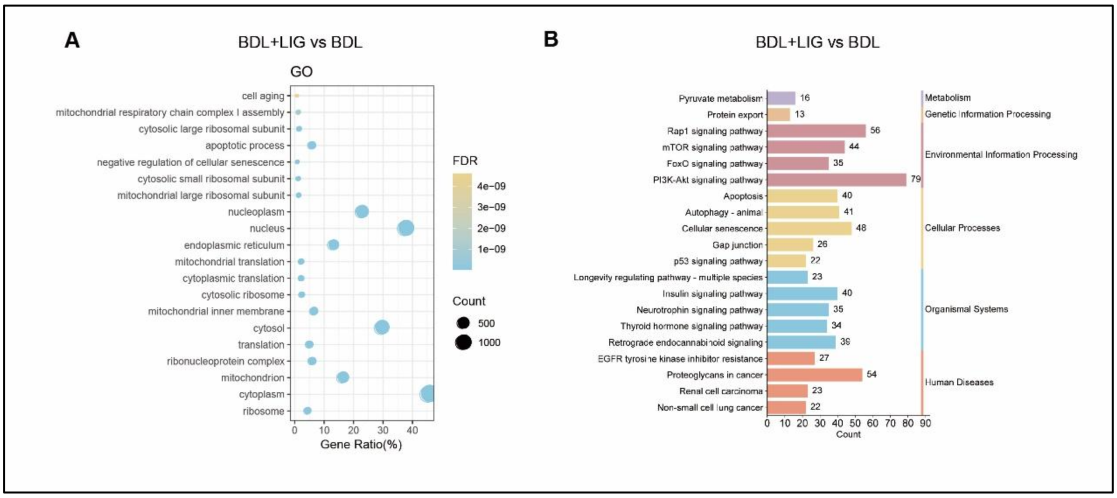

Figure S6. RNA-seq analysis based on BDL-induced fibrosis model. (**A**) GO analysis of BDL + LIG group vs BDL group. (**B**) KEGG analysis of BDL + LIG group and BDL group.

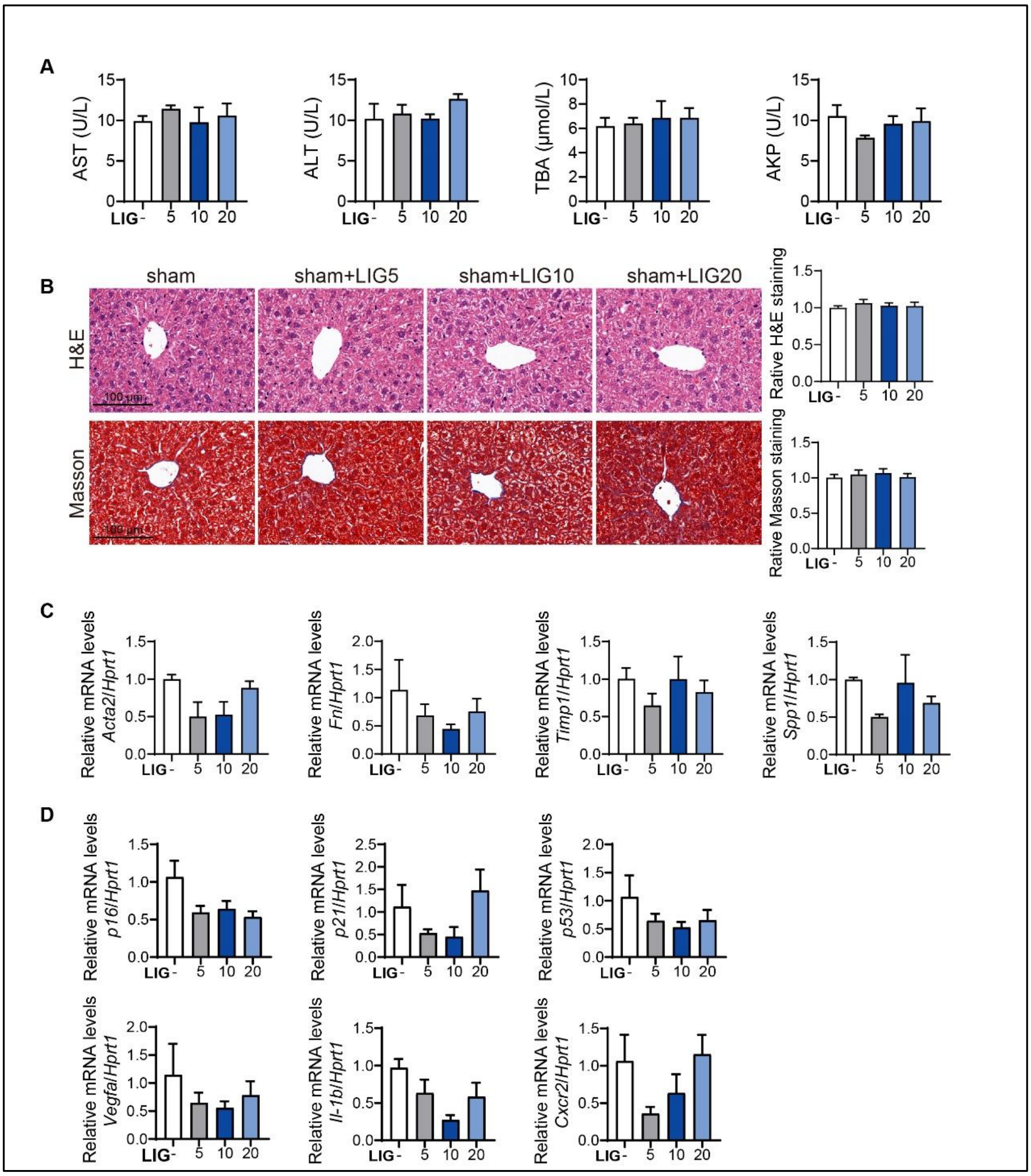

Figure S7. The effects of LIG treatment alone were evaluated *in vivo*. (**A**) Serum levels. (**B**) Representative images of H&E and Masson staining. The quantification results are shown in the right. (**C**) Relative mRNA levels of *Acta2*, *Fn1*, *Timp1*, and *Spp1* were assessed by qPCR and normalized to *Hprt1*. (**D**) Relative mRNA levels of senescence-related genes were determined by qPCR and normalized to *Hprt1*.

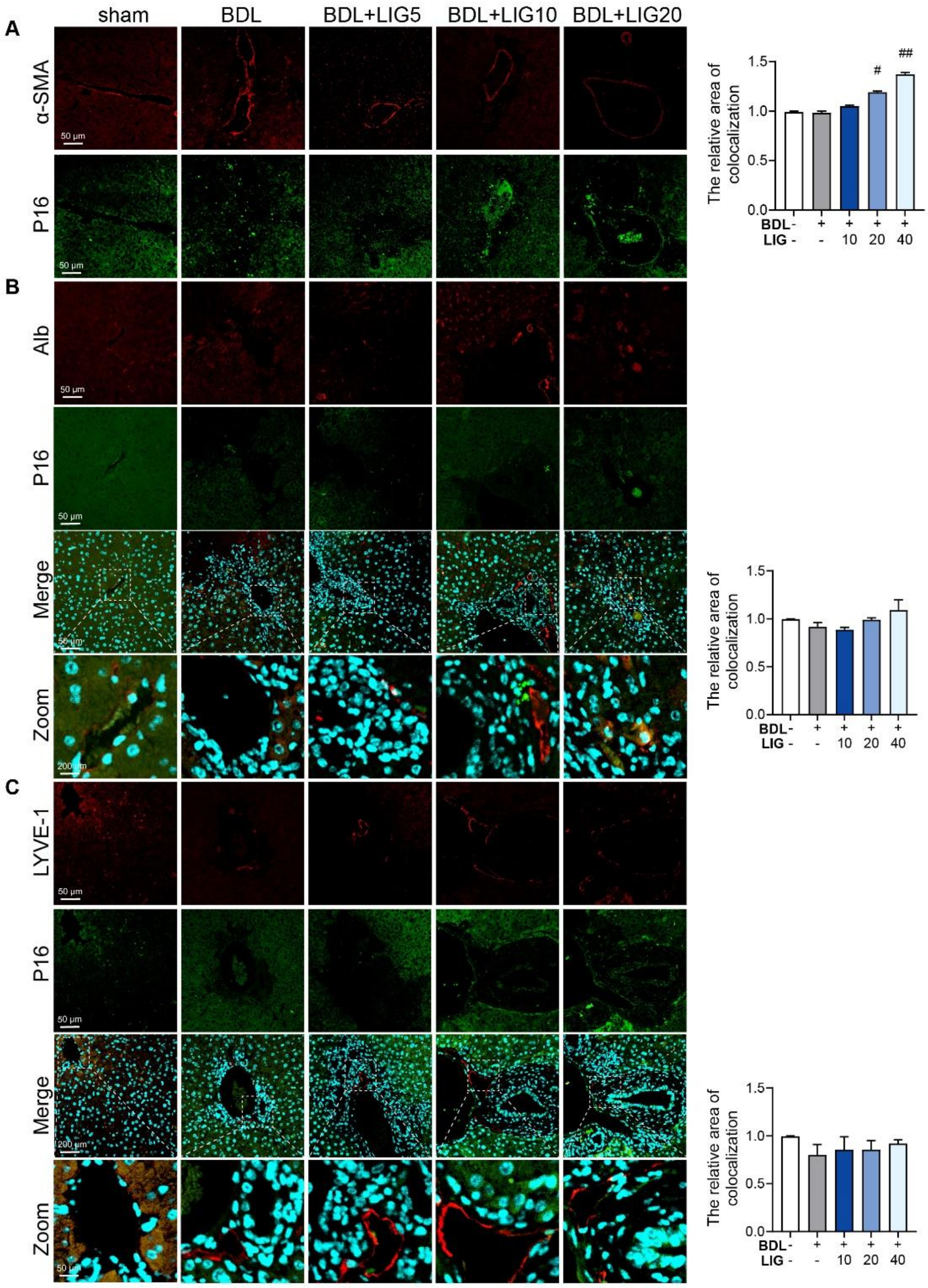

Figure S8. The senescence-inducing effect of LIG on different cell types in the BDL mice model. (**A**) Representative images of the staining of α-SMA (red) and P16 (green). (**B**) IF images of the co-staining of Alb (red), P16 (green) and DAPI (cyan). (**C**) IF images of the co-staining of LYVE-1 (red), P16 (green) and DAPI (cyan). The quantification of the IF results is presented on the right. Statistical significance: $^{\#}P < 0.05$, $^{\#\#}P < 0.01$, compared with the BDL group.

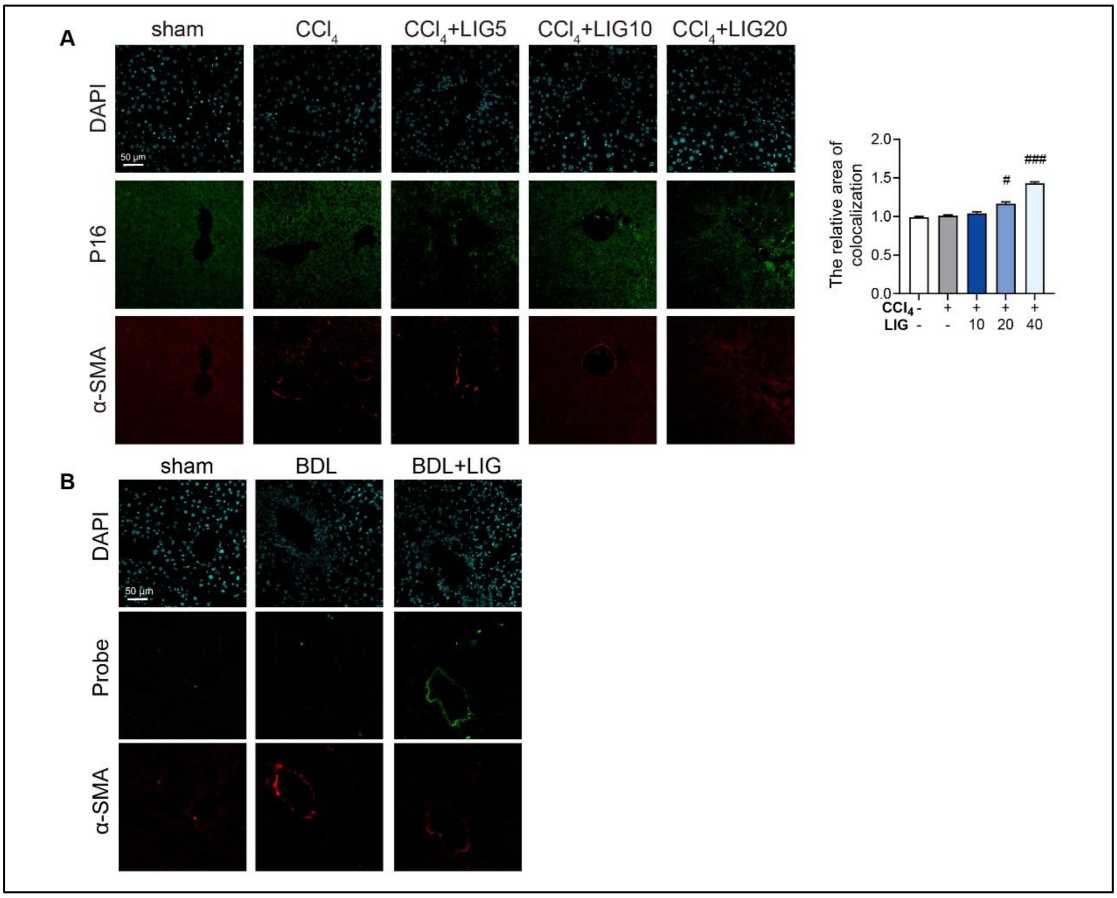

Figure S9. The senescence-inducing effect of LIG on HSC *in vivo*. (**A**) Representative images of the staining of α-SMA (red), P16(green) and DAPI (cyan). The quantification of the IF results is presented on the right. (**B**) Representative images of the staining of α-SMA (red), Senescence probe (green) and DAPI (cyan). Statistical significance: # $P < 0.05$, ### $P < 0.001$, compared with the CCl_4 group.

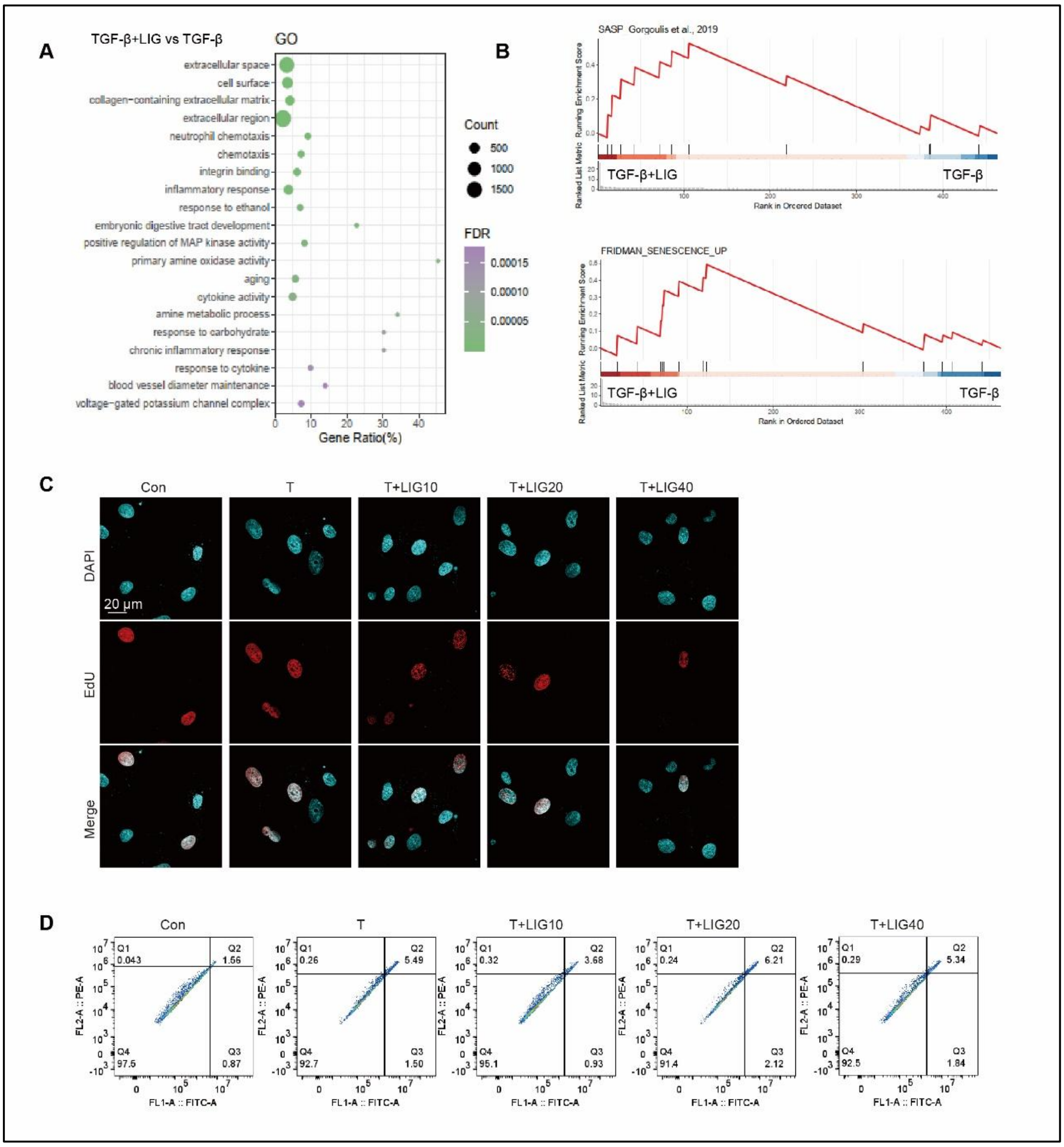

Figure S10. LIG promotes cell senescence in LX-2 cells. (**A**) GO analysis of DEGs between the TGF-β + LIG group and TGF-β group. **(B**) GSEA analysis of senescence-related pathway dependent on the cellular RNA-seq analysis. (**C**) EdU staining in LX-2 cells. **(D**) Annexin V/PI assay in LX-2.

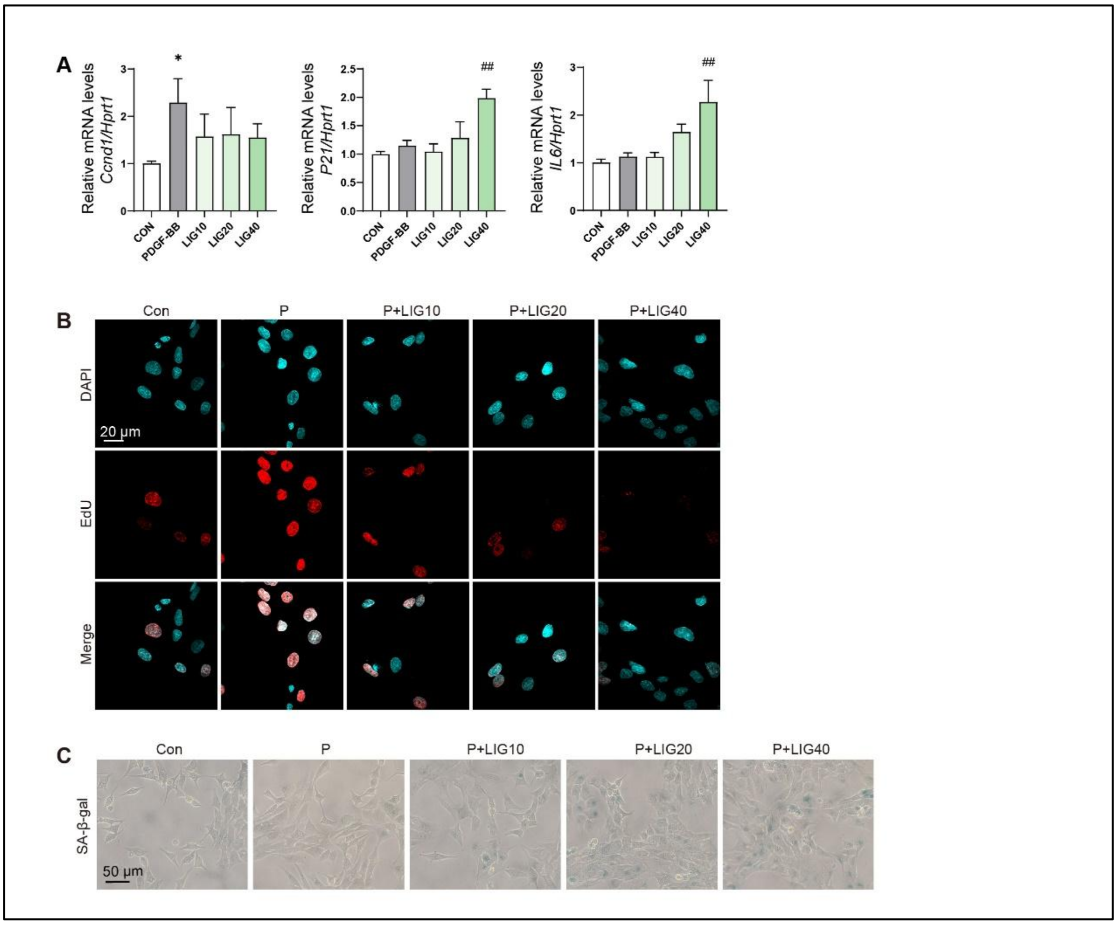

Figure S11. LIG promotes cell senescence in HSC-T6 cells. (**A**) Relative mRNA levels of *Ccnd1*, *p21* and *Il6* were determined in HSC-T6 cells by qPCR and normalized with *Hprt1*. (**B**) EdU staining in HSC-T6 cells. (**C**) SA-β-gal staining in HSC-T6 cells.

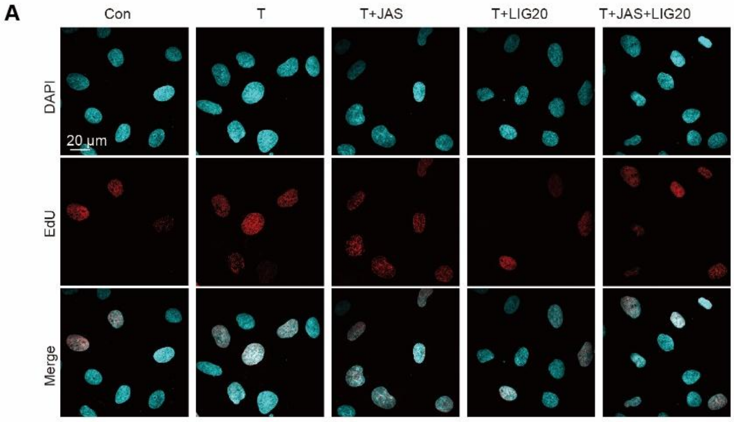

Fig. S12.The effects of JAS on the proliferation of LX-2 cells. (**A**) EdU staining in LX-2 cells.

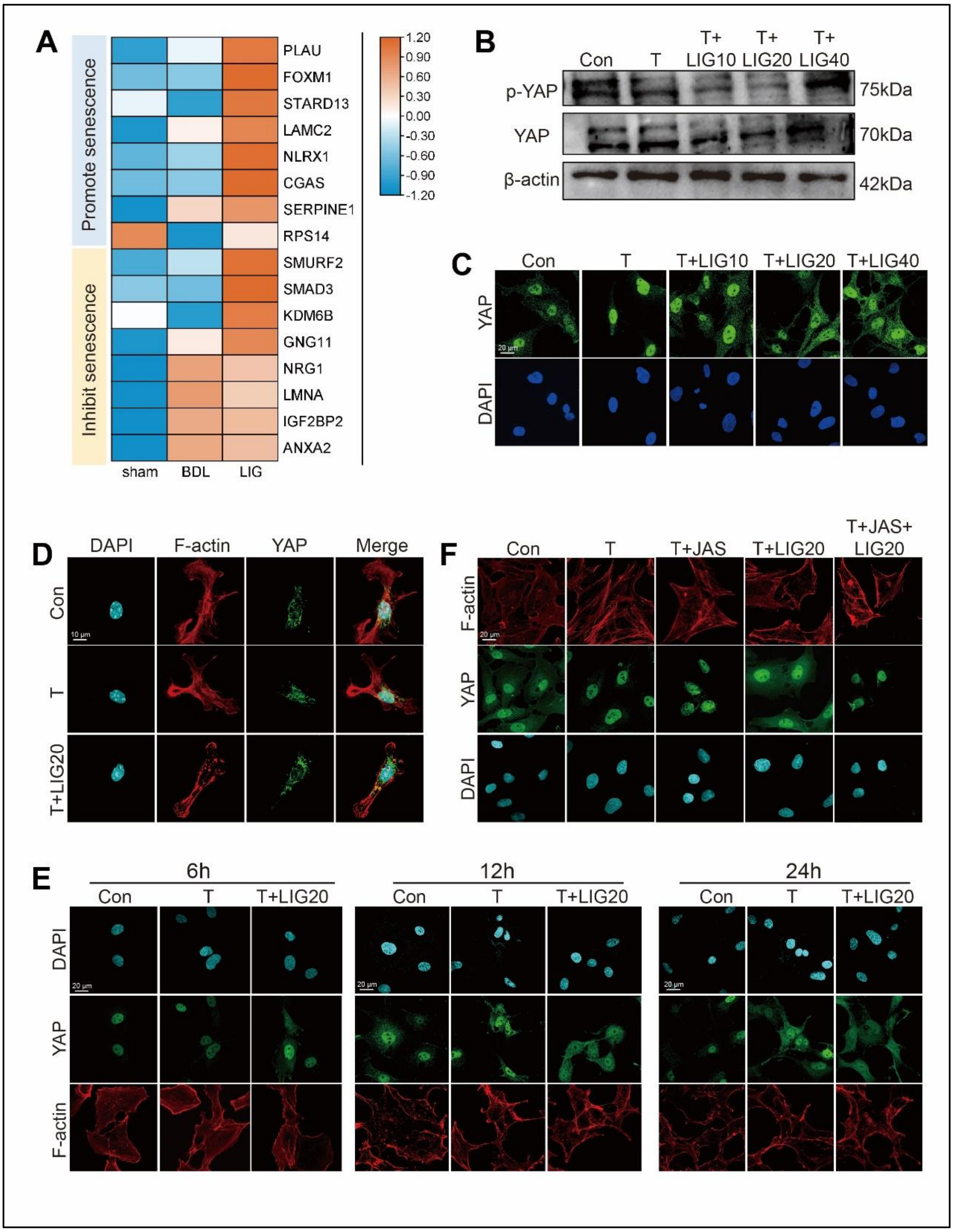

Figure S13. LIG inhibits YAP nucleoplasmic translocation *in vitro*. (**A**) The heatmap of YAP target genes associated with senescence. (**B**) The protein levels of YAP and p-YAP in LX-2 cells were detected by WB. (**C**) Representative images of IF staining for YAP (green) and DAPI (cyan) in LX-2 cells. (**D**) Representative images of IF staining for YAP (green), F-actin (red) and DAPI (cyan) in primary HSCs. (**E**) Representative images of IF staining for YAP (green), F-actin (red) and DAPI (cyan) in

LX-2 cells that treated with TGF-β or TGF-β + LIG (20 μM) for 6 h, 12 h or 24 h. (**F**) Representative images of IF staining for YAP (green), F-actin (red) and DAPI (cyan) after treating with JAS.

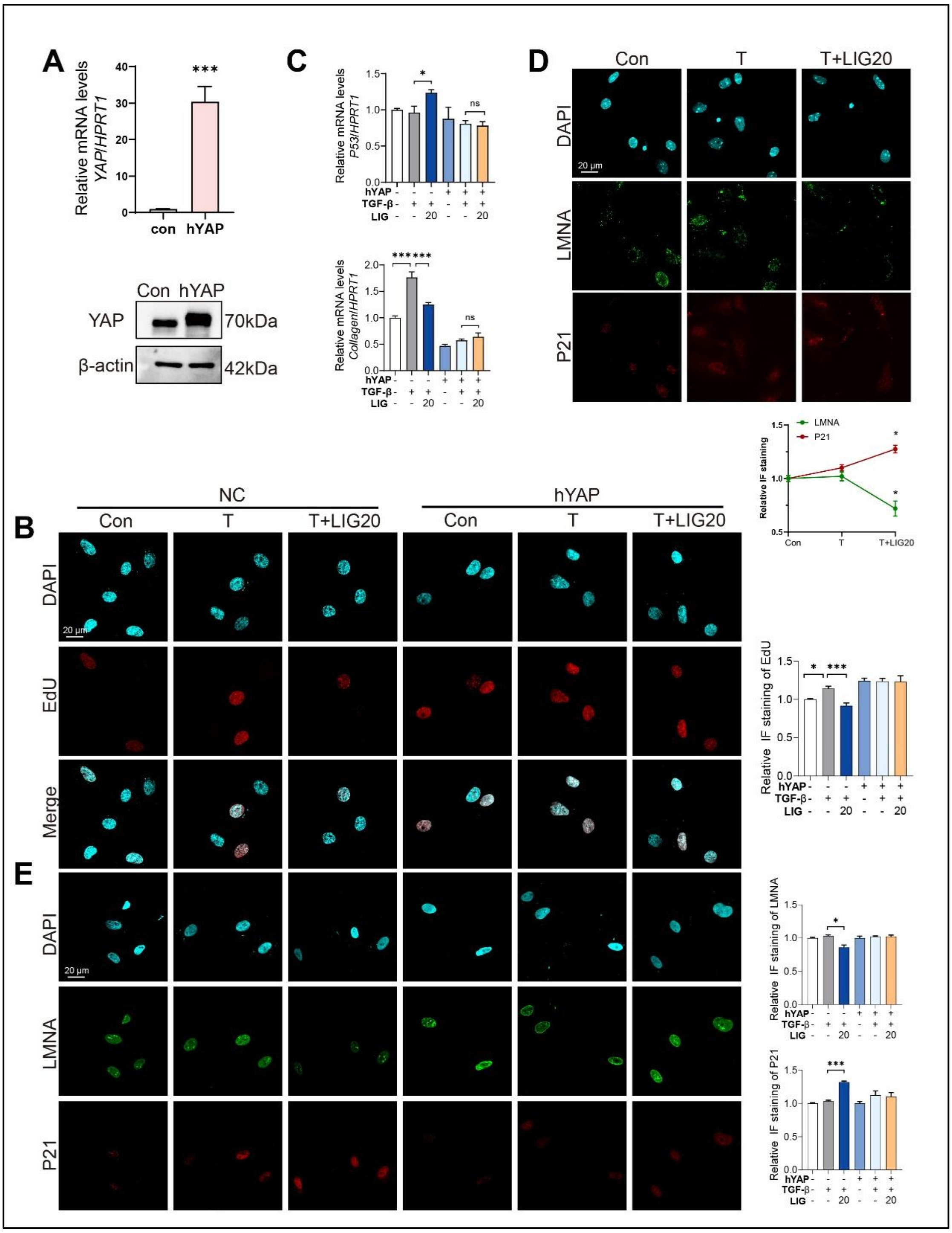

Figure S14. The overexpression of YAP reverses the inhibition of LIG *in vitro* (**A**) The overexpression level of YAP was verified by qPCR and WB in LX-2 cells. (**B**) Representative images of EdU staining in LX-2 cells with ct or OE-YAP plasmid transfection in LX-2 cells. (**C**) Relative mRNA levels of *P53* and *Collagen* were determined by qPCR and normalized with *HPRT1* after the ct or OE-hYAP plasmid transfection. (**D**) Representative images of IF staining for LMNA (green), P21 (red) and

DAPI (cyan) in primary HSCs. (**E**) Representative images of IF staining for LMNA (green), P21 (red) and DAPI (cyan) after the ct or OE-YAP plasmid transfection in LX-2 cells. The quantification of the IF results is presented beside. Statistical significance: * $P < 0.05$, *** $P < 0.001$.

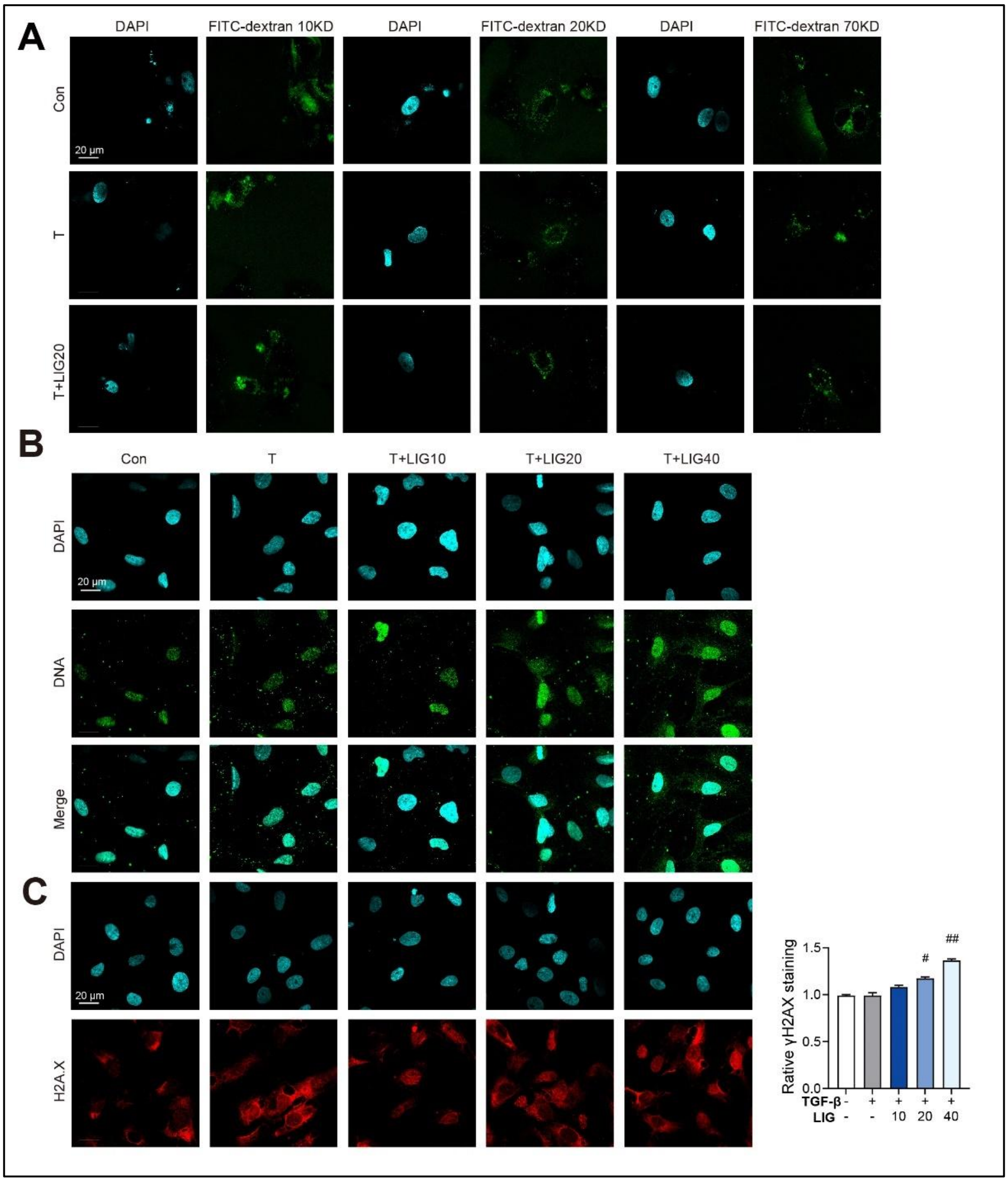

Figure S15. LIG affects the nuclear envelope integrity and DNA leakage. (**A**) Representative images of IF staining for FITC-Dextran (green) and DAPI (cyan) images. (**B**) Representative images of IF staining for DNA (green) and DAPI (cyan) in LX-2 cells. (**C**) Representative images of IF staining for γH2A.X (red) and DAPI (cyan) in LX-2 cells. The quantification of the IF results is presented on the right. Statistical significance: # $P < 0.05$, ## $P < 0.01$, compared with the TGF-β group.

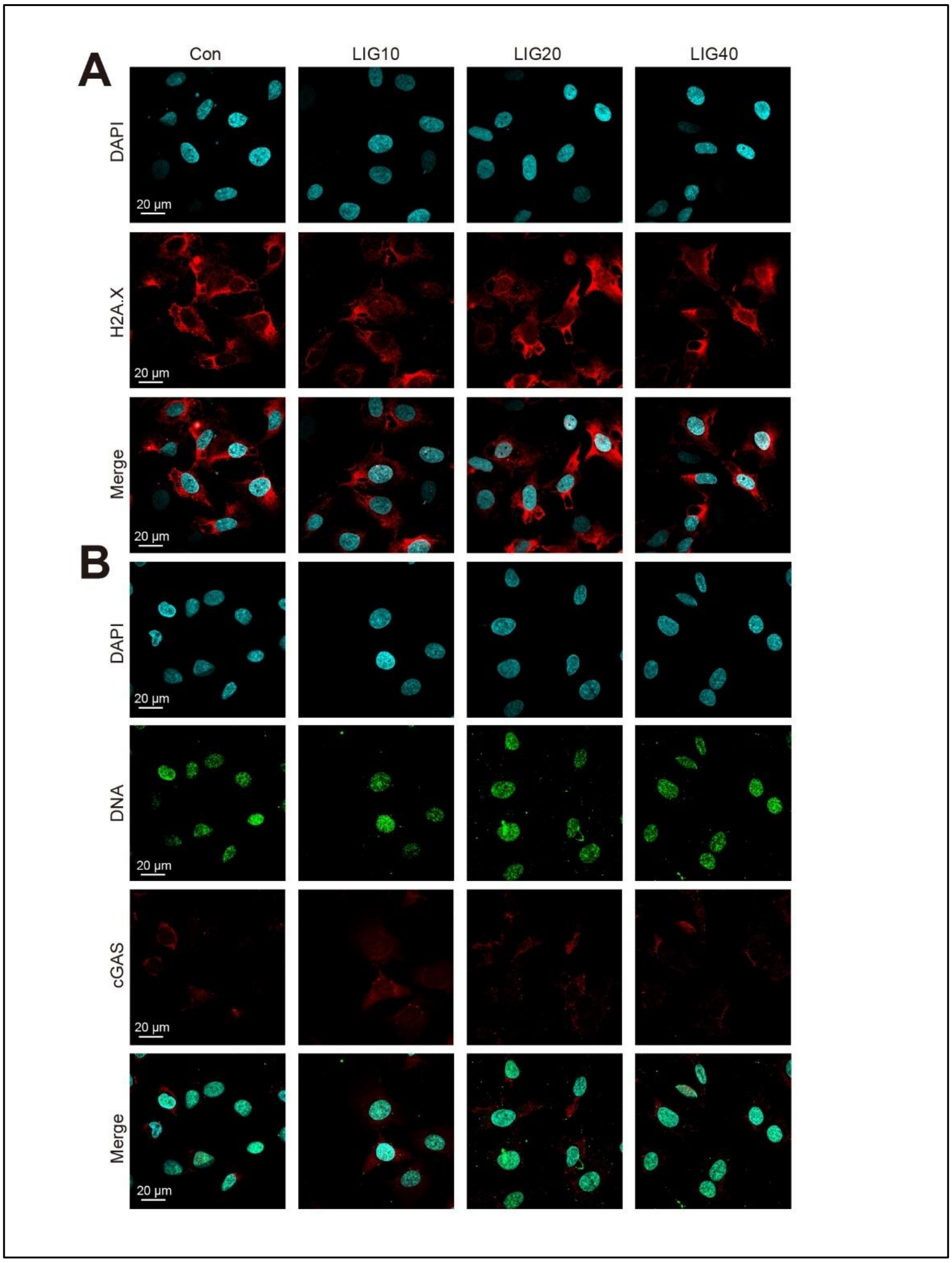

Figure S16. The modulation of LIG treatment alone on the dsDNA-cGAS-STING pathway. (**A**) IF co-staining of γH2A.X (red) and DAPI (cyan) in LX-2 cells. (**B**) IF co-staining of cGAS (red), DNA (green) and DAPI (cyan) in LX-2 cells.

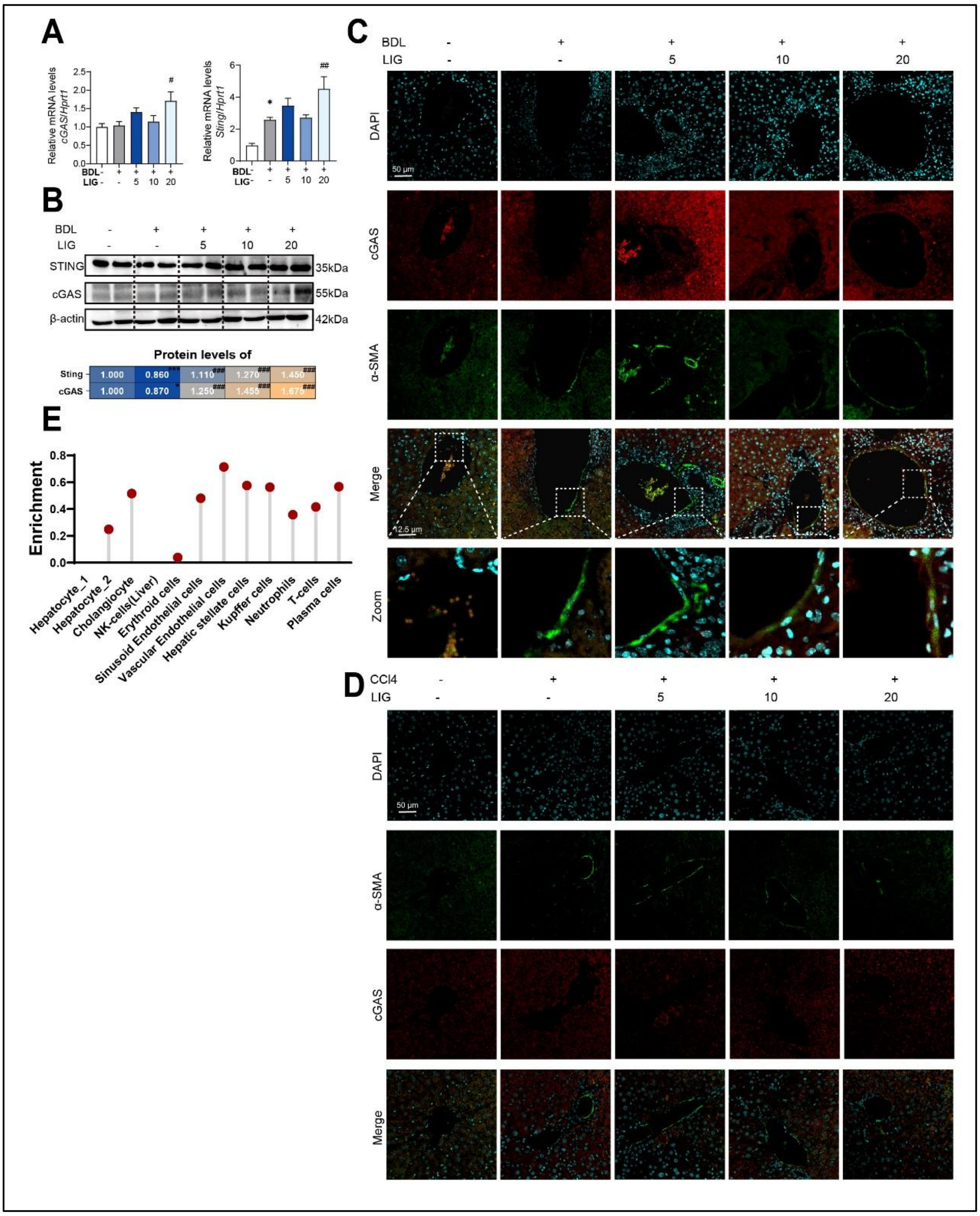

Figure S17. LIG activated the cGAS-STING pathway *in vivo*. (**A**) Relative mRNA levels of *cGAS* and *Sting* in BDL model were determined by qPCR and normalized with *Hprt1*. (**B**) The protein levels of cGAS and STING from liver tissues in BDL model were determined by WB and normalized by β-actin. (**C-D**) Representative images of IF staining for cGAS (red), α-SMA (green) and DAPI (cyan) in mice liver tissue from BDL model (**C**) and CCl_4 model (**D**). (**E**) The relative enrichment of STING in hepatic cells. Statistical significance: * $P < 0.05$, compared with sham group; # $P < 0.05$, ## $P <$

0.01, $^{###}$ $P < 0.001$, compared with the BDL group.

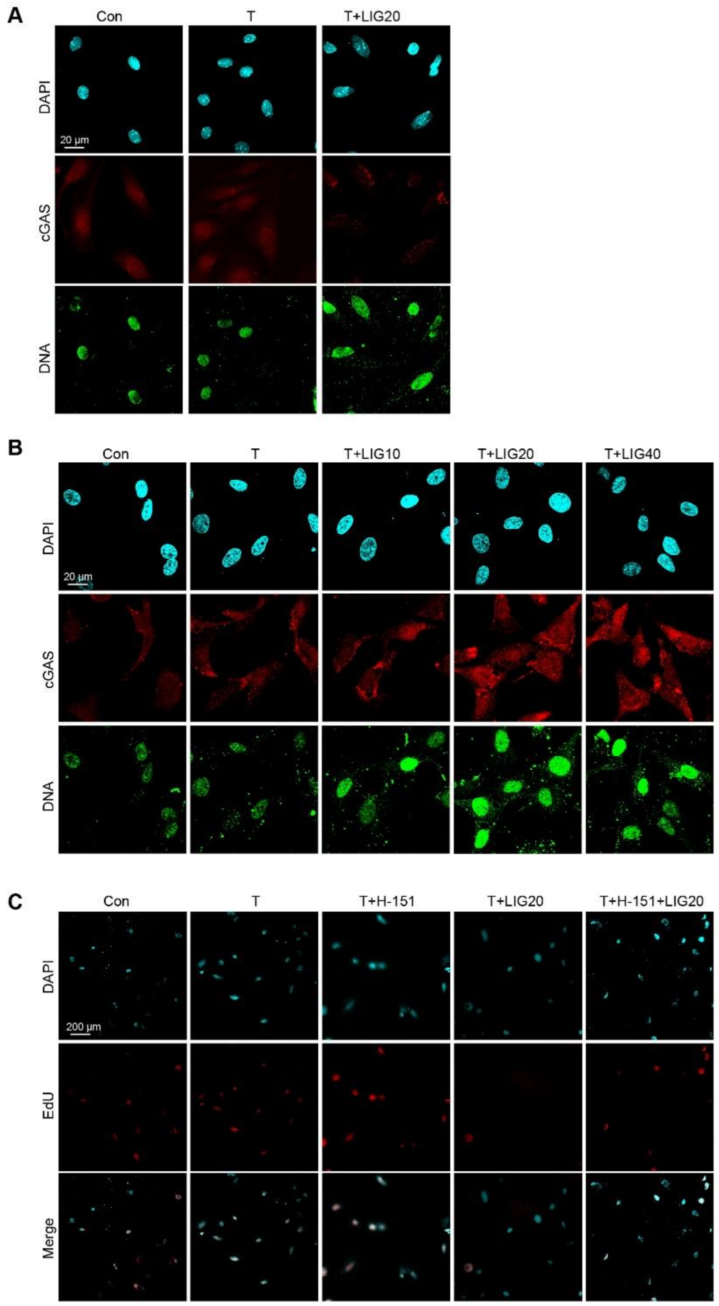

Figure S18. The cGAS-STING pathway is involved in LIG-induced senescence *in vitro*. (**A-B**) Representative images of IF staining for cGAS (red), DNA (green) and DAPI (cyan) in primary HSCs (**A**) and LX-2 cells (**B**). (**C**) Representative images of EdU staining in LX-2 cells with H-151 treatment.

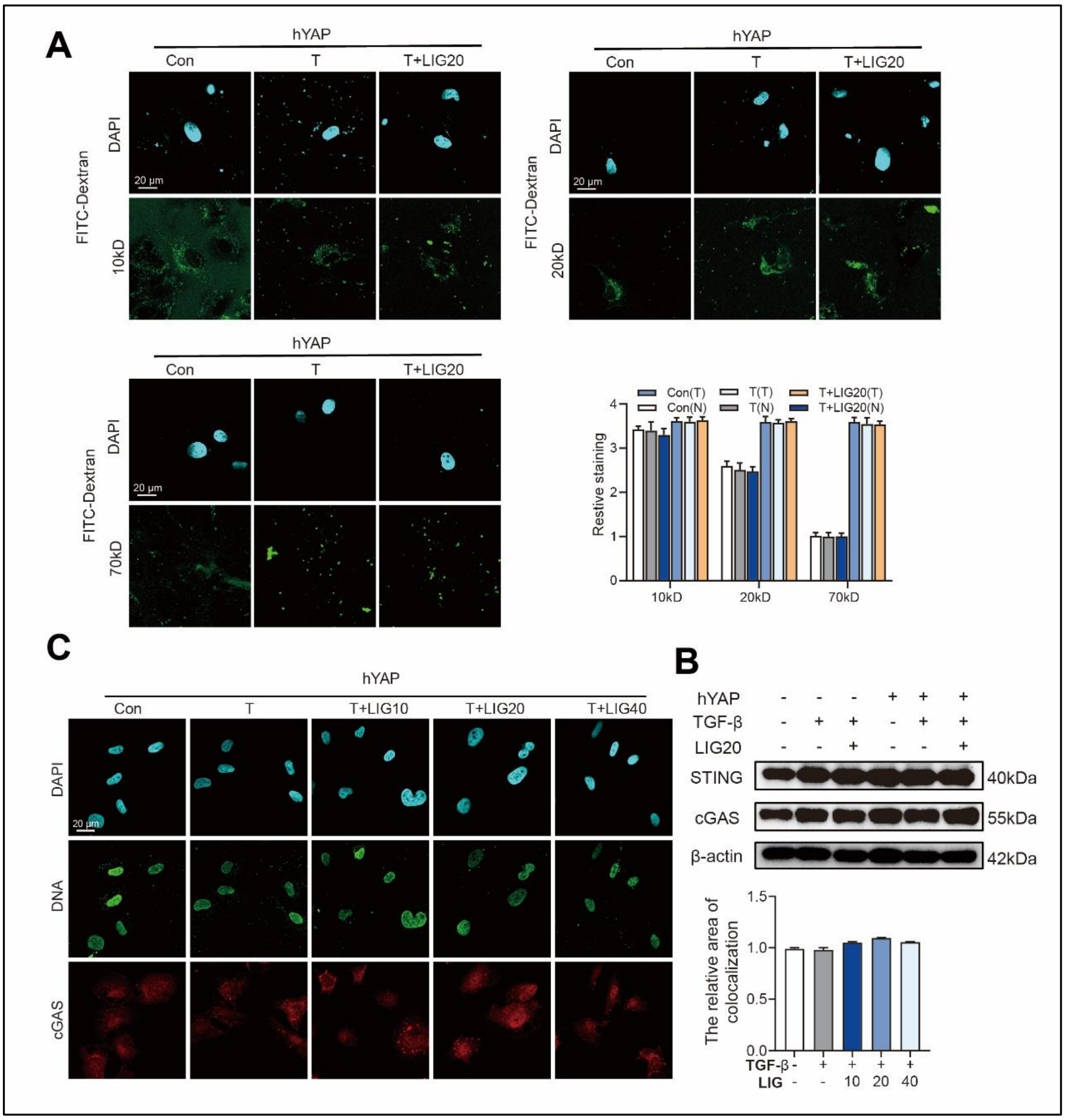

Figure S19. YAP overexpression reveres the effects of LIG on the integrity of the nuclear membrane and the regulation of STING and cGAS. (**A**) Representative images of FITC-Dextran (green) and DAPI (cyan). (**B**) The protein levels of cGAS and STING were determined by WB and normalized by β-actin. (**C**) Representative images of IF staining for cGAS (red), DNA (green) and DAPI (cyan) in LX-2 cells after the plasmid transfection. The quantification of the IF results is presented beside.

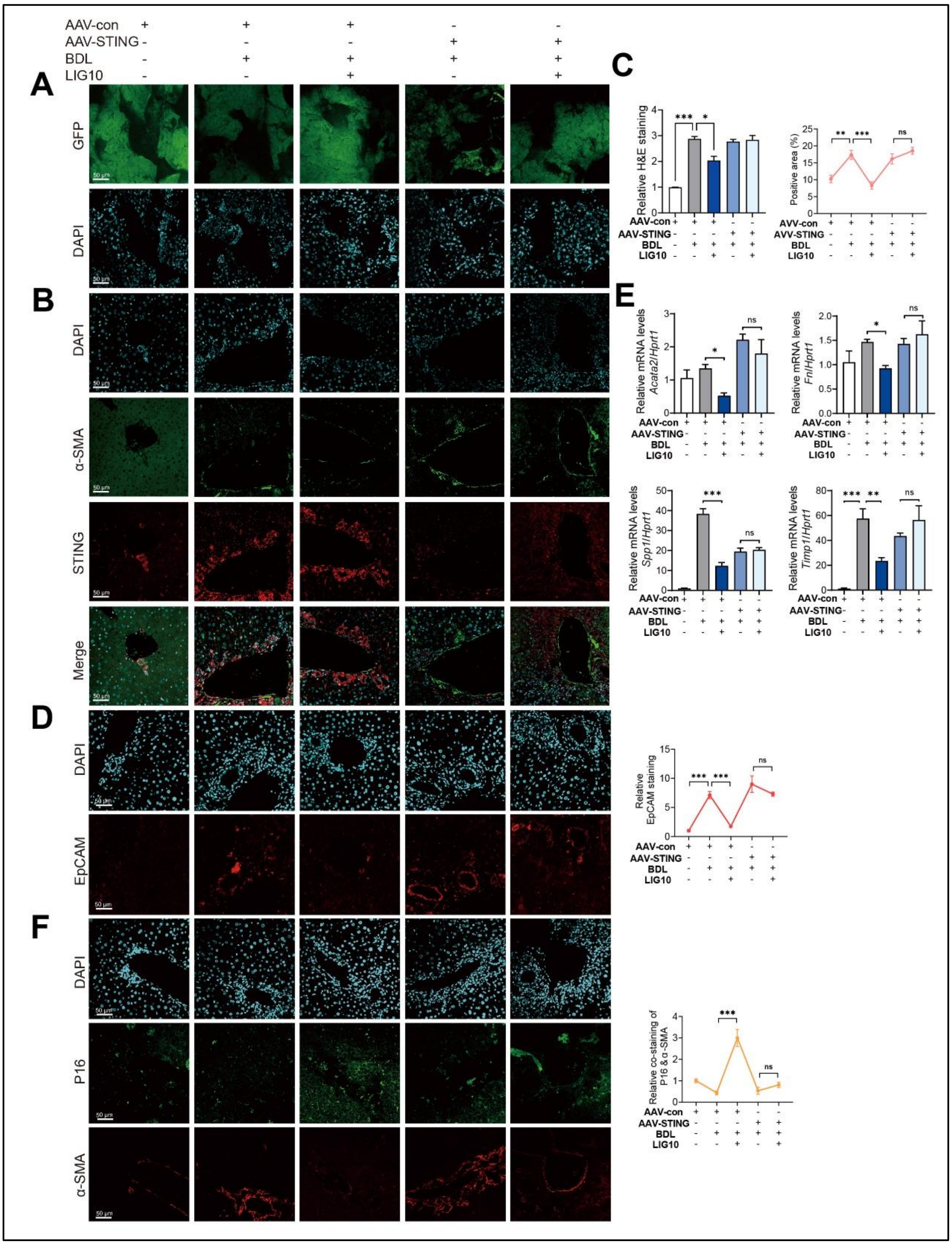

Figure S20. Knockdown of STING in mice can eliminate the *in vivo* anti-fibrotic effects of LIG. (**A**) The staining of GFP and DAPI from AAV8-shSTING mice liver tissues. (**B**) IF co-staining images of α-SMA (green), STING (red), DAPI (cyan). (**C**) Statistical results of H&E and Sirius red. (**D**) IF staining images of EpCAM (red) and DAPI (cyan). (**E**) Relative mRNA levels of *Acta2*, *Fn, Spp1* and *Timp1* were determined

by qPCR and normalized with *Hprt1*. (**F**) IF co-staining images of α-SMA (red), P16 (green) and DAPI (cyan). The quantification of the IF results is presented on the right. Statistical significance: * $P < 0.05$, ** $P < 0.01$, *** $P < 0.001$.

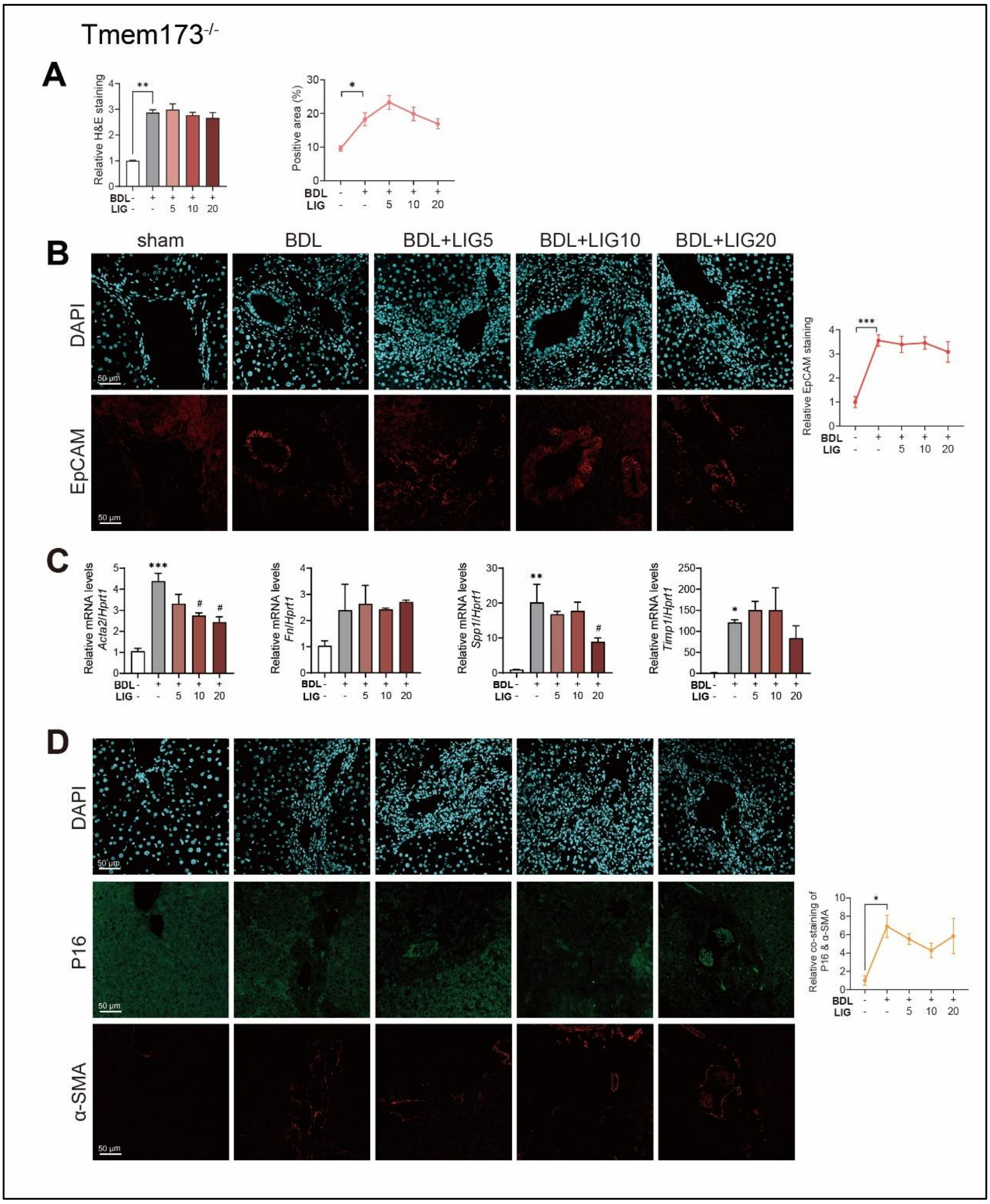

Figure S21. Knockout of STING in mice can eliminate the *in vivo* anti-fibrotic effects of LIG. (**A**) Statistical results of H&E and Sirius red. (**B**) Representative staining images of EpCAM (red) and DAPI (cyan) from STING KO mice liver tissues. (**C**) Relative mRNA levels of *Acta2*, *Fn, Spp1* and *Timp1* were determined by qPCR and normalized with *Hprt1*. (**D**) IF co-staining images of α-SMA (red), P16 (green) and DAPI (cyan). Statistical significance: * P < 0.05, ** P < 0.01, *** P < 0.001, compared with sham group; # P < 0.05, compared with the BDL group.

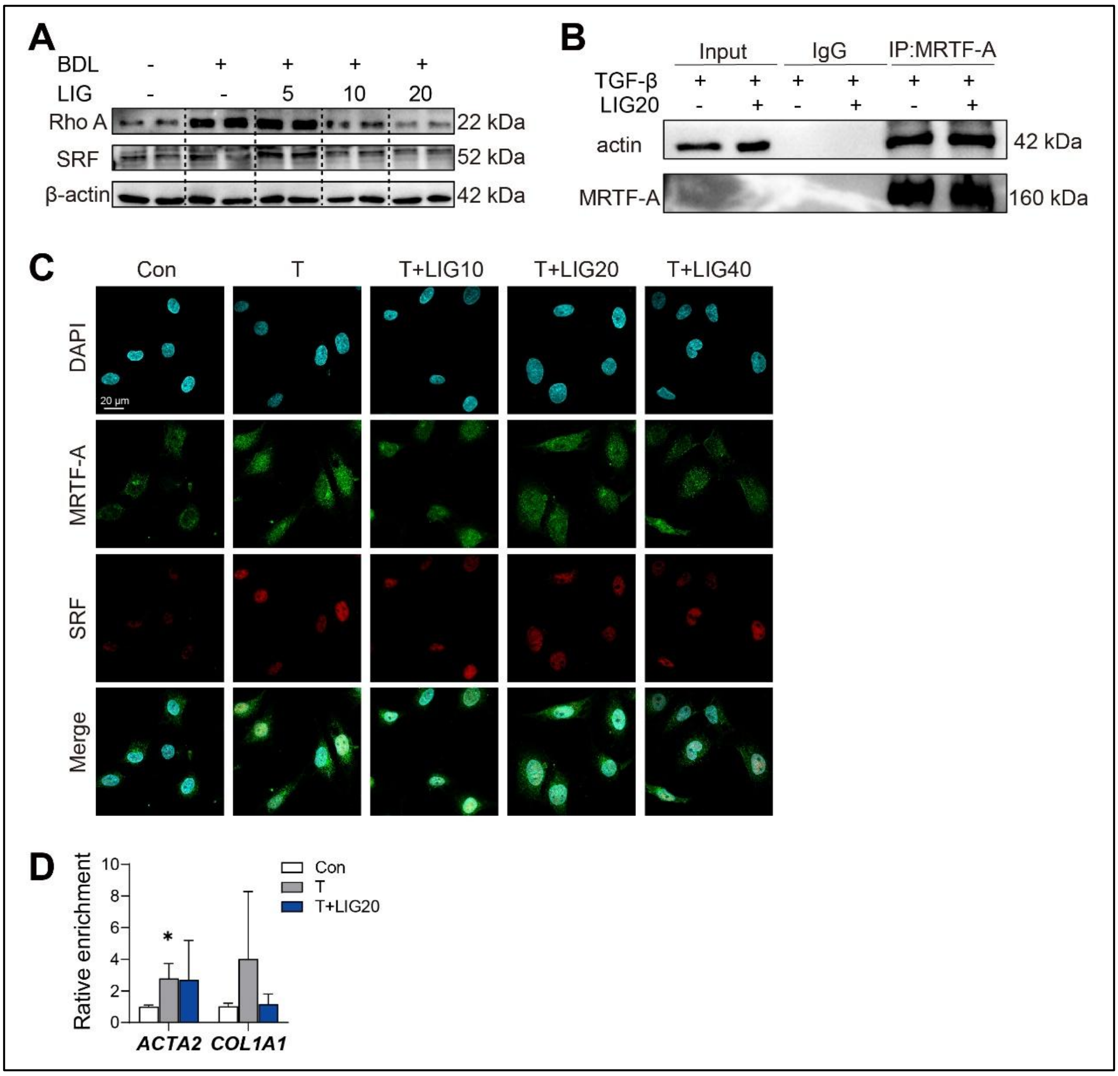

Figure S22. The effects of LIG on the Rho-MRTF-A-SRF pathway. (**A**) The protein levels of RhoA and SRF were determined by WB and normalized by β-actin. (**B**) IP analysis of the combination of actin and MRTF-A. (**C**) IF co-staining of SRF (red), MRTF-A (green) and DAPI (cyan). (**D**) ChIP-qPCR analysis for SRF. * P < 0.05, compared with the Con group.
